# Optimizing Exercise Prescriptions for Cognitive Subdomains in Diabetes: A Systematic Review and Meta-Analysis of Dose–Response Variables

**DOI:** 10.3390/bs16071218

**Published:** 2026-07-18

**Authors:** Qin Sun, Yong He, Huanyu Wang, Jieping Wang, Yu Feng, Haili Tian, Yue Feng

**Affiliations:** 1 College of Physical Education and Health Science, Zhejiang Normal University, Jinhua 321004, China; sq950316@zjnu.edu.cn (Q.S.);; 2 School of Exercise and Health, Shanghai University of Sport, Shanghai 200438, China; 3 Center for Translational Medicine, Naval Medical University, Shanghai 200433, China

**Keywords:** diabetes, cognitive function, exercise intervention, exercise variables

## Abstract

**Objective:** This systematic review and meta-analysis aimed to evaluate the effects of exercise interventions on cognitive function in diabetes. **Methods:** We conducted a comprehensive search of the Web of Science, PubMed, Scopus, EMBASE (Ovid), and SPORTDiscus databases from inception to December 2025 (registration number: CRD420251046731). The review included randomized controlled trials focusing on exercise interventions for diabetes and their impact on 4 cognitive domains: executive function, memory, attention, and global cognition. Subgroup and moderator analyses were performed based on exercise variables, including exercise type, session duration, total weekly exercise time, and intervention period. Study quality was assessed using the Cochrane Risk of Bias Tool 2 and the Physical Therapy Evidence Database scale (PEDro scale). **Results:** After screening 6230 records, 16 studies were included. Exercise interventions produced small-to-medium significant improvements in memory and global cognition in patients with diabetes, whereas the overall pooled effect on executive function was not statistically significant. Subgroup analyses suggested potential trends toward improvement in executive function for aerobic exercise (Hedge’s *g* = −0.30, *p* = 0.0039), single sessions ≥ 45 min (Hedge’s *g* = −0.40, *p* = 0.0463), and intervention periods ≥ 24 weeks (Hedge’s *g* = −0.27, *p* = 0.0383). For memory, aerobic (Hedge’s *g* = 0.27, *p* = 0.0131) or multicomponent exercise (Hedge’s *g* = 0.45, *p* = 0.0224) with single sessions ≤ 60 min (Hedge’s *g* = 0.28, *p* = 0.002), total weekly exercise time ≤ 120 min (Hedge’s *g* = 0.35, *p* = 0.0012), and interventions ≥ 12 weeks (Hedge’s *g* = 0.32, *p* = 0.0003) were significantly beneficial. Exercise also had a significant positive effect on global cognition (Hedge’s *g* = 0.49, *p* < 0.0001) without significant differences across exercise variables. In addition, exercise intervention showed a borderline, non-significant trend toward improved motor performance in patients with diabetes (Hedge’s *g* = 0.33, *p* = 0.0506). **Conclusions:** This comprehensive meta-analysis supports the beneficial effects of exercise on global cognition and memory in patients with diabetes. For memory enhancement, aerobic or multicomponent exercise protocols of ≥12 weeks, ≤120 min of total weekly exercise, and ≤60 min per session are more recommended. Although the overall pooled effect on executive function did not reach statistical significance, exploratory subgroup findings point toward potential domain-specific benefits under targeted exercise parameters (such as aerobic exercise of longer duration). These dose–response patterns should therefore be viewed as exploratory and hypothesis-generating, warranting confirmation in adequately powered, head-to-head trials before specific exercise prescriptions can be recommended.

## 1. Introduction

Diabetes, a major global metabolic disease, currently affects approximately 537 million individuals worldwide, imposing severe health burdens (H. Sun et al., 2022). Epidemiological studies show that the global diabetic population will increase to 783.2 million (12.2% of the global population) by 2045 (H. Sun et al., 2022). One notable complication of diabetes is cognitive dysfunction, which has been increasingly recognized as a comorbidity (Antal et al., 2022; Biessels & Despa, 2018; Xue et al., 2019; You et al., 2021). Chronic hyperglycemia and insulin resistance damage the central nervous system through mechanisms such as neuronal injury, disrupted neural signaling, and cerebral blood flow abnormalities, contributing to cognitive decline and neurodegenerative diseases (Ehtewish et al., 2022; Ji et al., 2023). Studies show that diabetes increases the risk of cognitive impairment and dementia by 1.25–1.9 times (with a relative risk of 1.53), with cognitive dysfunction prevalence reaching 42.9% (Xue et al., 2019; Zheng et al., 2023). Cognitive impairment has a negative impact on cognitive domains such as executive function, memory, and attention in diabetic patients (Cooke et al., 2020; Xiong et al., 2021). These cognitive domains are related to numerous aspects of daily life, including quality of life and work ability, and they can serve as effective assessment tools for predicting physical activity levels, physical impairment, and mortality (Vazzana et al., 2010; J. Zhang et al., 2024). Despite the known prevalence of cognitive dysfunction in diabetes, current therapeutic and preventive strategies remain underdeveloped (de Galan et al., 2009; Dolan et al., 2018; Ji et al., 2023; Luo et al., 2022).

Over the past several decades, numerous clinical and retrospective studies have shown that physical exercise can improve cognitive function to varying extents in specific populations, including those with age-related cognitive decline, Alzheimer’s disease, and attention-deficit hyperactivity disorder (Boa Sorte Silva et al., 2024; Brini et al., 2018; Kuo et al., 2024; Ornish et al., 2024; M. Zhang et al., 2023). Furthermore, physical exercise is a critical component of diabetes management, offering effective non-pharmacological therapeutic benefits at a low cost (Kirwan et al., 2017). The American College of Sports Medicine has highlighted the positive effects of regular physical activity on both psychological well-being and cognitive function in individuals with diabetes (Kanaley et al., 2022). However, the evidence specifically addressing the impact of physical exercise on cognitive function in diabetic patients remains limited and inconclusive. For example, a clinical trial investigating the cognitive effects of physical exercise in adults with and without diabetes found that a 24-month multicomponent exercise intervention significantly improved global cognition and memory in diabetic patients, but had no significant impact on executive function. Conversely, Leischik et al. (Espeland et al., 2017) reported that a 3-month walking intervention led to significant improvements in executive function among diabetic patients, whereas verbal and nonverbal memory outcomes varied—potentially due to differences in exercise volume (Leischik et al., 2021). Additionally, an 8-year randomized controlled trial revealed that increased physical activity failed to significantly influence cognitive function in obesity with type 2 diabetes mellitus (Espeland et al., 2014). Given the current evidence, a comprehensive investigation is warranted to elucidate the cognitive benefits of physical exercise in individuals with diabetes, resolve existing discrepancies, and optimize clinical practice.

The American College of Sports Medicine recommends that patients with diabetes engage in long-term physical exercise to manage their condition and reduce associated health risks (Kanaley et al., 2022). Specifically, when clinical professionals consider exercise prescriptions to prevent or mitigate cognitive decline, they need to clarify the effects of exercise variables, including duration, frequency, intensity, and type of exercise, on various cognitive domains (Kanaley et al., 2022; M. Zhang et al., 2023). As previously mentioned, accurate implementation of these variables may effectively improve cognitive function in diabetic patients. On the other hand, there remains a lack of research information on exercise prescriptions for managing cognitive functions in diabetic patients. While recent meta-analyses suggest that exercise benefits cognitive functions (e.g., global cognition) in diabetic patients, these studies suffer from experimental and methodological limitations. For example, they involve a limited number of included studies or single-moderator comparisons (Lu et al., 2024; Z. Sun et al., 2024; R. Wang et al., 2021; J. Zhang et al., 2024). These limitations hinder a comprehensive understanding of how exercise affects cognitive performance in diabetic patients, particularly with respect to the domain-specific effects of different exercise parameters.

This systematic review and meta-analysis aim to comprehensively evaluate the effects of physical exercise on cognitive function in patients with diabetes. Additionally, subgroup analyses will further investigate the impacts of exercise variables (i.e., type, duration, and intervention period) on multiple cognitive domains. These findings are intended to provide evidence-based guidance for clinicians and patients, supporting the development of individualized exercise interventions for cognitive health.

## 2. Method

### 2.1. Overview

The meta-analysis was conducted in accordance with the Preferred Reporting Items for PRISMA guidelines and the Cochrane Collaboration Handbook (Cumpston et al., 2019). A literature search was performed to identify relevant studies published up to December 2025 across 5 databases: Web of Science, PubMed, Scopus, EMBASE (Ovid), and SPORTDiscus. In addition, this study has been registered with the International Prospective Register for Systematic Reviews (registration number: CRD420251046731). The checklist see Table S5 (Page et al., 2021). An amalgamation of MeSH and non-MeSH terms were employed (Supplementary Table S4). Additional eligible studies were identified through supplementary approaches, including hand-searching and reviewing reference lists.

### 2.2. Inclusion and Exclusion Criteria

The selected studies were included according to the Population, Intervention, Comparison, Outcomes, and Study model (Table 1) (Q. Sun et al., 2024; Xiong et al., 2021; J. Zhang et al., 2024). Intervention studies conducting exercise therapy were included. To minimize methodological heterogeneity and ensure a high degree of comparability among the included studies, we restricted inclusion to clinical trials. Studies were excluded if (a) research data were not available and (b) the full-text data and detailed results were insufficient.

### 2.3. Data Extraction and Coding

The preliminary review of the records from all databases and the eligibility of the studies was conducted by 2 separate investigators (H.Y.W. and Y.F.). Then, the results were confirmed by another 2 separate investigators (J.P.W. and Q.S.), and any disagreements regarding the data were resolved through discussion with a third investigator (H.L.T.). After removing duplicates, all remaining articles were filtered by title and abstract (stage 1) and then by full-text specific content (stage 2). Records were imported into Endnote (Version X9.3.3; Clarivate Analytic, Philadelphia, PA, USA) and we performed automated and manual screening. After finalizing the screening of the included studies, the data were categorized based on participant characteristics (sample size, age, and years) and intervention type. Exercise variables were coded according to the American College of Sports Medicine guidelines and prior research: exercise type, single-session duration, total weekly exercise time, and intervention period (F. T. Chen et al., 2020; Ding et al., 2025; Kanaley et al., 2022; M. Zhang et al., 2023). For the assessment of cognitive function, we included cognitive subdomains such as executive function, memory, attention, and overall cognition. Evaluating these subdomains to represent cognitive function is necessary, as general neurocognitive performance is clinically characterized by these interconnected subdomains (Ding et al., 2025; Harvey, 2019; M. Zhang et al., 2023). Details of the cognitive assessment tools and motor performance measures used in the included studies are provided in Supplementary Table S2 (Ding et al., 2025; M. Zhang et al., 2023).

### 2.4. Quality and Risk of Bias Assessments

The methodological quality of each eligible randomized controlled trials was assessed by two independent investigators (Q.S. and Y.F.) using the Cochrane Risk of Bias Tool 2 (RoB 2) tool (Sterne et al., 2019) and PEDro scale (Verhagen et al., 1998). Disagreements were resolved through consultation with a third investigator (H.L.T.), who helped reconcile the differences. The RoB 2 assessment covered five key domains: (1) randomization process, (2) deviations from intended interventions, (3) missing outcome data, (4) measurement of outcomes, and (5) selection of reported results. Studies were evaluated using the RoB 2 tool and classified into three categories: “low risk of bias” (when all assessment domains met the low-risk criteria), “some concerns” (if at least one domain raised concerns), and “high risk of bias” (defined by at least one high-risk domain or multiple domains with concerns). For each study, a total score of 0–10 was determined. Studies were quality-rated as low (PEDro ≤ 3), moderate (4–5), or high (≥6). All were included in data synthesis irrespective of methodological quality.

### 2.5. Statistical Analysis

All meta-analytic calculations were performed using R software (version 4.4.2) with the “meta” and “metafor” packages (Schwarzer et al., 2015; Viechtbauer, 2010). For each outcome, the meta-analytic model was selected according to the degree of statistical heterogeneity: a fixed-(common-) effect model was applied when heterogeneity was low (*I*^2^ < 49%), whereas a random-effects model was applied when heterogeneity was moderate or high (*I*^2^ ≥ 50%) to account for the greater between-study variability; forest plots were generated to visualize the results (Veroniki et al., 2016). Effect sizes (Hedges’ *g* with 95% CIs) for executive function differences between the exercise and control groups were computed based on post- intervention means and standard deviations (SDs). For reported intervention effects with dispersion measures (e.g., standard error, 95% CIs), we estimated parameters using established guidelines (Higgins et al., 2019). In studies featuring shared control groups, we divided the sample size of the control group by two to prevent unit-of-analysis errors (Xiong et al., 2021). The magnitude of the effect size was categorized as small (0.2 ≤ g < 0.5), medium (0.5 ≤ g < 0.8), or large (g ≥ 0.8), with statistical significance at α < 0.05 (two-tailed). The results of a minimum of three studies had to be used to create quantitative synthesis. Study heterogeneity was assessed using *I*^2^ statistics (low: <49%, moderate: 50–74%, and high: >74%) (F. T. Chen et al., 2020). Subgroup analyses were conducted to determine potential moderating effects of exercise frequency, exercise type, session duration, total exercise time per week, and intervention length on executive function outcomes. In order to explore potential sources of heterogeneity, we performed meta-regression analysis with session duration, total exercise time per week and intervention length. To assess robustness, we conducted a sensitivity analysis restricted to studies in which executive function was a prespecified primary cognitive outcome, and compared primary- versus secondary-outcome studies using a test for subgroup differences.

## 3. Results

### 3.1. Literature Search

**Figure 1:**
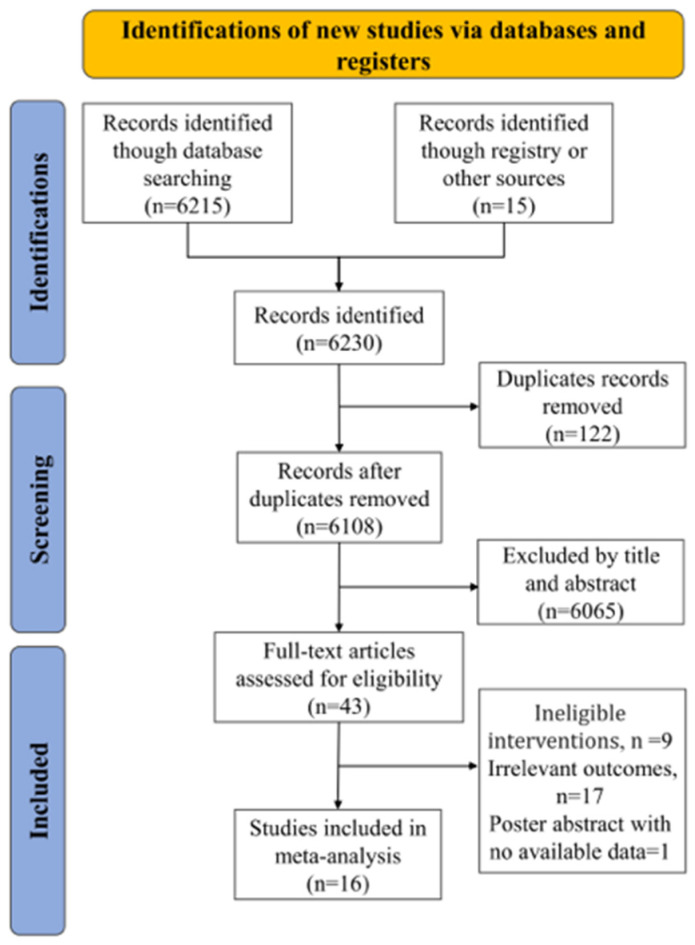
The Preferred Reporting Items for Systematic Reviews and Meta-analyses (PRISMA) flow diagram of the literature search.

A total of 16 eligible studies were included for quantitative analysis after screening. As shown in Figure 1, a total of 6230 intervention studies were retrieved from five databases (Web of Science, PubMed, Scopus, EMBASE [Ovid], and SPORTDiscus) and relevant literature sources. During the screening phase (including duplicate articles, titles, and abstracts), 6182 duplicate or irrelevant articles were excluded. Twenty-seven articles were excluded from this meta-analysis for the following reasons: (a) ineligible intervention, (b) irrelevant outcome measures, and (c) poster abstracts with no available data.

### 3.2. Study Characteristics

Sixteen eligible RCTs were included in the current quantitative analysis after screening. Characteristics of the included studies are shown in Supplementary Table S1. The participants of the study were all type 2 diabetes mellitus patients, except for one study with type 1 diabetes mellitus (Suh et al., 2019). Except for one study, participants had no cognitive impairments at the baseline. In terms of the subdomains of cognitive function, the most was executive function (n = 10) (Callisaya et al., 2017; Y. Chen et al., 2023; Furlano et al., 2023; Ghahfarrokhi et al., 2024; Liu et al., 2024; Plotnikoff et al., 2010; Ploydang et al., 2023; Silveira-Rodrigues et al., 2021; Yanagawa et al., 2011; Zhao et al., 2022), which was followed by memory (n = 8) (Callisaya et al., 2017; Y. Chen et al., 2023; Furlano et al., 2023; Liu et al., 2024; Shellington et al., 2018; Silveira-Rodrigues et al., 2021; Suh et al., 2019; Zhao et al., 2022), global cognition (n = 7) (Y. Chen et al., 2023; Ghahfarrokhi et al., 2024; Ghodrati et al., 2023; Molina-Sotomayor et al., 2020; W. Sun et al., 2025; Y. Wang et al., 2023; Yanagawa et al., 2011), and attention (n = 4) (Y. Chen et al., 2023; Ghodrati et al., 2023; Shellington et al., 2018; Silveira-Rodrigues et al., 2021). Across the 16 included studies (a total of 1102 people), experimental group interventions utilized diverse physical exercise. The most common type of exercise was aerobic exercise (n = 8) (Y. Chen et al., 2023; Liu et al., 2024; Molina-Sotomayor et al., 2020; Ploydang et al., 2023; Shellington et al., 2018; W. Sun et al., 2025; Y. Wang et al., 2023; Yanagawa et al., 2011), followed by multicomponent physical exercise (n = 5) (Callisaya et al., 2017; Ghahfarrokhi et al., 2024; Ghodrati et al., 2023; Silveira-Rodrigues et al., 2021; Suh et al., 2019), and resistance exercise (n = 3) (Furlano et al., 2023; Plotnikoff et al., 2010; Zhao et al., 2022). Physical exercise was typically performed 3 times weekly, with session durations ranging from 22 to 120 min and intervention periods spanning 6 to 48 weeks. Of the included studies, the physical exercise intensity was controlled at 50–70% of maximum heart rate (HR_max_) in 1 study (Y. Chen et al., 2023), 40–65% of maximum oxygen consumption (VO_2max_) in 1 study (Molina-Sotomayor et al., 2020), 80% of 1 repetition maximum in 2 studies (Furlano et al., 2023; Zhao et al., 2022), the range of Borg Scale rating of perceived well-being (RPE) 12–13 in 1 study (Callisaya et al., 2017), and 40–60% of heart rate reserve in 2 studies (Liu et al., 2024; Ploydang et al., 2023), but the exercise intensity was not stated in the remaining 9 studies. None of the included studies reported related adverse events.

### 3.3. Methodological Quality and Risk of Bias

**Figure 2:**
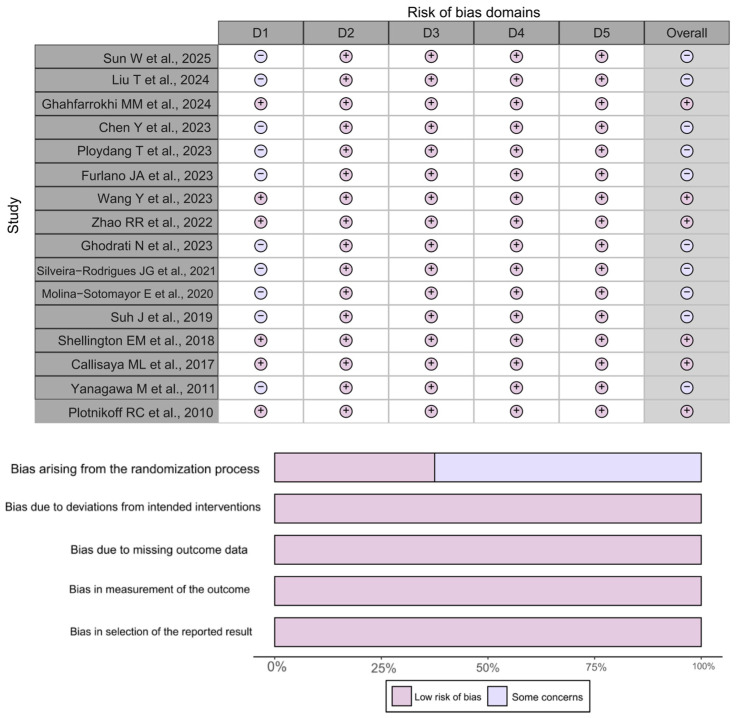
The risk of bias assessment for the included studies was conducted using the Cochrane RoB2 tool across five domains. Risk levels are indicated as low (pink), unclear (light purple), and high (dark purple) (Callisaya et al., 2017; Y. Chen et al., 2023; Furlano et al., 2023; Ghahfarrokhi et al., 2024; Ghodrati et al., 2023; Liu et al., 2024; Molina-Sotomayor et al., 2020; Plotnikoff et al., 2010; Ploydang et al., 2023; Shellington et al., 2018; Silveira-Rodrigues et al., 2021; Suh et al., 2019; W. Sun et al., 2025; Y. Wang et al., 2023; Yanagawa et al., 2011; Zhao et al., 2022). D1: Bias due to randomization; D2: Bias due to deviations from intended intervention; D3: Bias due to missing data; D4: Bias due to outcome measurement; D5: Bias due to selection of reported result; +: Low; −: Some concerns.

The methodological quality of the included 16 randomized controlled trials evaluated using the Cochrane RoB2 tool is presented in Figure 2. Of these, 10 studies demonstrated an overall low risk of bias, while 6 studies raised some concerns, primarily regarding randomization procedures. This mainly occurred when it came to the participation of the subjects and their allocation to the intervention measures, with the allocation sequence either being concealed or there being no information available.

In addition, the methodological quality of the included studies was generally high (mean PEDro scale score = 6, range = 3–8). Eleven out of the 16 randomized controlled trials were considered to be of good quality (total score 6–8), 4 were moderate, and the remaining studies were deemed poor quality (score ≤ 3) (Supplementary Table S3).

### 3.4. Effect Measures

#### 3.4.1. Executive Function

**Figure 3:**
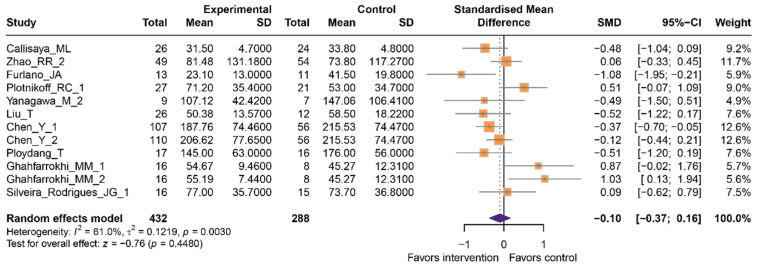
Forest plot of physical exercise in executive function; 95%CI = 95% confidence interval; *I*^2^ = inconsistency between studies; SMD = standardized mean difference.

Ten studies (involving 720 participants) reported the effects of exercise on overall executive function in patients with diabetes. Given the diversity of outcome measures, Hedges’ *g* values were used to analyze the pooled effects. Preliminary analysis showed that exercise interventions did not significantly improve executive function in diabetic patients (Hedge’s *g* = −0.10, 95% CI = −0.37–0.16, *p* > 0.05, *I*^2^ = 61%, Figure 3). To explore potential moderators and generate future hypotheses, we conducted exploratory subgroup analyses across exercise type, session duration, weekly exercise time, and intervention period (Table 2): (1) A potential trend toward improvement in executive function was observed within the aerobic exercise subgroup (Hedge’s *g* = −0.30, 95% CI = −0.50–−0.10, *p* = 0.0039, *I*^2^ = 0%, Figure S1); (2) Compared with single-session durations < 45 min (Hedge’s *g* = 0.13, 95% CI = −0.28–0.53, *p* = 0.5368, *I*^2^ = 68.2%, Figure S2), sessions of ≥45 min indicated an exploratory trend toward positive effects (Hedge’s *g* = −0.40, 95% CI = −0.80–−0.01, *p* = 0.0463, *I*^2^ = 45.7%, Figure S2); (3) A total weekly exercise duration of 120 min did not effectively alter executive function (Figure S3); (4) Interventions period ≥ 24 weeks also showed potential positive effects (Hedge’s *g* = −0.27, 95% CI = −0.52–−0.01, *p* = 0.0383, *I*^2^ = 48.7%, Figure S4).

#### 3.4.2. Memory

**Figure 4:**
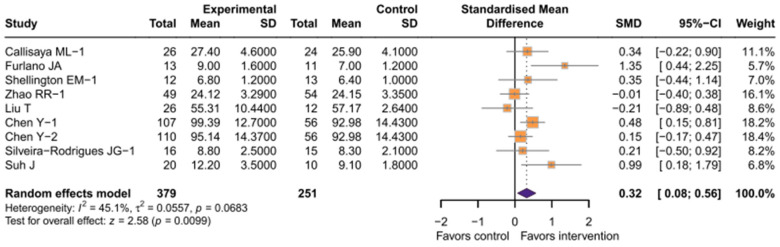
Forest plot of physical exercise in memory; 95%CI = 95% confidence interval; *df* = degree of freedom; *I*^2^ = inconsistency between studies; SMD = standardized mean difference.

Eight studies (involving 630 participants) evaluated the effects of exercise on memory in patients with diabetes. Pooled analysis showed that physical exercise significantly improved memory (Hedge’s *g* = 0.32, 95% CI = 0.08–0.56, *p* = 0.0099, *I*^2^ = 45.1%, Figure 4). Subgroup analyses (Table 3) revealed significant positive effects for aerobic exercise (Hedge’s *g* = 0.27, 95% CI = 0.06–0.48, *p* = 0.0131, *I*^2^ = 25%, Figure S5) and multicomponent exercise (Hedge’s *g* = 0.45, 95% CI = 0.06–0.83, *p* = 0.0224, *I*^2^ = 13.1%, Figure S5). Further analysis indicated that physical exercise sessions of ≤60 min (Hedge’s *g* = 0.28, 95% CI = 0.10–0.45, *p* = 0.002, *I*^2^ = 58.6%, Figure S6), total weekly exercise durations of ≤120 min (Hedge’s *g* = 0.35, 95% CI = 0.14–0.56, *p* = 0.0012, *I*^2^ = 35.3%, Figure S7), and intervention period ≥ 12 weeks (Hedge’s *g* = 0.32, 95% CI = 0.15–0.49, *p* = 0.0003, *I*^2^ = 51.5%, Figure S8) all significantly improved memory in diabetic patients.

#### 3.4.3. Attention

**Figure 5:**
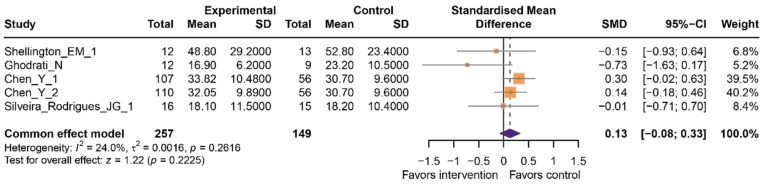
Forest plot of physical exercise in attention; 95%CI = 95% confidence interval; *df* = degree of freedom; *I*^2^ = inconsistency between studies; SMD = standardized mean difference.

Four studies (involving 406 participants) reported on attention. Compared with control groups, physical exercise had no significant effect on attention in patients with diabetes (Figure 5, Table 4).

#### 3.4.4. Global Cognition

**Figure 6:**
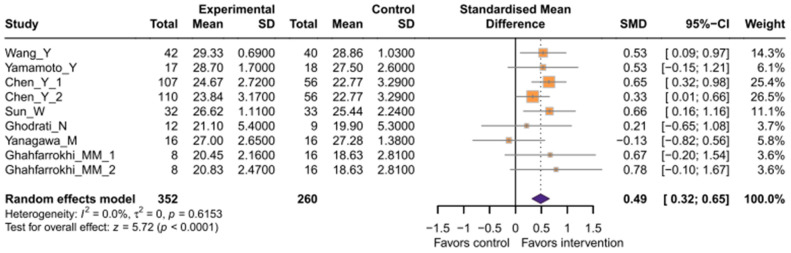
Forest plot of physical exercise in global cognition; 95%CI = 95% confidence interval; *df* = degree of freedom; *I*^2^ = inconsistency between studies; SMD = standardized mean difference.

Seven studies (involving 612 participants) evaluated the effects of physical exercise on global cognition in patients with diabetes. Overall, exercise interventions significantly improved global cognition (Hedge’s *g* = 0.49, 95% CI = 0.32–0.65, *p* < 0.0001, *I*^2^ = 0%, Figure 6). Subgroup analyses (Table 5) revealed no significant differences across type of exercise (aerobic and multicomponent exercise), exercise duration, or intervention period, with all subgroups demonstrating significant improvements in global cognition (Figures S13–S16).

### 3.5. Potential Moderators

**Figure 7:**
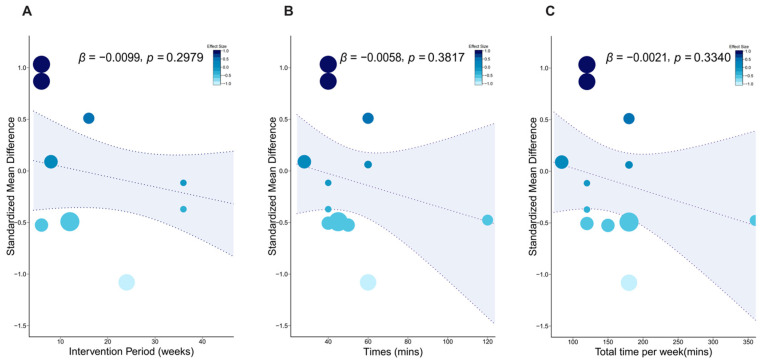
Meta-regression analyses of exercise variables and cognitive sub-domains. (**A**) Meta-regression analysis of intervention period and executive function. (**B**) Meta-regression analysis of single-session exercise duration and executive function. (**C**) Meta-regression analysis of total weekly exercise duration and executive function.

Meta-regression was used to assess the effects of single-session exercise duration, weekly total exercise time, and total intervention period on the effect sizes. The analysis showed that the factors did not significantly modulate executive function (Figure 7). In addition, these indicators showed a negative trend with SMD. Meanwhile, intervention duration tended to be positively associated with improvements in memory and global cognition, and single exercise duration and total weekly exercise time tended to be positively associated with improvements in attention or global cognition (Figures S17–S19).

### 3.6. Motor Performance

Four studies (involving 153 participants) investigated the effect of exercise on motor function in patients with diabetes. The results showed that exercise intervention was associated with a borderline, non-significant trend toward improved motor performance in patients with diabetes (Hedge’s *g* = 0.33, 95% CI = −0.00–0.66, *p* = 0.0506, *I*^2^ = 46.6%, Figure S20). In addition, subgroup analysis revealed that multicomponent exercise (Hedge’s *g* = 0.94, 95% CI = 0.31–1.58, *p* = 0.0036, *I*^2^ = 0%, Figure S21) and intervention period < 12 weeks (Hedge’s *g* = 0.94, 95% CI = 0.31–1.58, *p* = 0.0036, *I*^2^ = 0%, Figure S22) had a significant positive effect on motor performance.

## 4. Discussion

This meta-analysis comprehensively evaluates the effects of different exercise variables on cognitive function and its subdomains in individuals with diabetes. From the perspective of exercise prescription, this study examined the moderating effects of exercise type, single exercise duration, total weekly exercise time and intervention period on cognitive function (including executive function, memory, attention, and global cognition). Overall, exercise interventions were associated with small to moderate improvements in memory and global cognition, whereas a statistically non-significant overall trend was observed for executive function. Notably, the type of exercise (aerobic exercise or multicomponent exercise), the duration of single exercise (45–60 min), and the total cycle (≥12 weeks) emerged as key moderators in improving cognitive function in individuals with diabetes. There has been no meta-analysis to study whether exercise training affects specific domains of cognitive function in patients with diabetes, which will help to provide parameters for formulating exercise prescriptions for the prevention and treatment of cognitive impairment in diabetes.

### 4.1. Executive Function

The primary pooled analysis of this study demonstrated that physical exercise interventions did not lead to a statistically significant overall improvement in executive function among patients with diabetes. This non-significant overall metric suggests that physical exercise, when pooled globally across heterogeneous types, intensities, and durations, does not consistently modulate executive control in this clinical population. Previous studies, however, indicate that the benefits of exercise on cognitive function in diabetic patients may be most pronounced in the domain of executive function (Gu et al., 2021). Further moderator analyses were conducted to explore the impact of exercise variables, such as exercise type and intervention duration. In terms of exercise type, subgroup analyses showed that aerobic exercise performed for ≥24 weeks and session of ≥45 min significantly improved executive function. These subgroup findings suggest that adjusting exercise variables related to aerobic exercise might modulate executive function in diabetes. This is consistent with 3 recent meta-analyses in healthy individuals, which also identified aerobic exercise as an effective intervention for improving executive function (F. T. Chen et al., 2020; Xiong et al., 2021; Zhao et al., 2018). However, given the non-significance of the overall primary analysis, these subgroup results must be interpreted with caution as exploratory and hypothesis-generating rather than confirmatory.

In fact, the positive effects of aerobic exercise on executive function have been demonstrated in both animal models and clinical trials. From a neurobiological perspective, aerobic exercise counteracts insulin resistance, restores hippocampal mitochondrial function, and regulates apoptotic processes, thereby helping to improve executive function (Cui et al., 2017; Park et al., 2018). Aerobic exercise also significantly increases brain-derived neurotrophic factors and other growth factors, which promote neuronal survival, synapse formation, and neuroplasticity, particularly in the prefrontal cortex (the core brain area for executive function) (Cotman & Berchtold, 2002). Moreover, cross-sectional and prospective brain imaging studies suggest that aerobic exercise reduces brain atrophy in older individuals, especially in regions supporting executive control and memory (S. J. Colcombe et al., 2006; Kramer et al., 2006).

Interestingly, compared with aerobic exercise, multicomponent and resistance exercises did not show distinct positive trends in subgroups. The mechanism behind this may be that multicomponent exercise, which combines two or more types of exercise, dilutes the specific benefits of aerobic exercise on executive function. A previous meta-analysis reported that multicomponent exercise yielded inferior cognitive benefits compared to the same duration (4 weeks) of 30-min aerobic exercise or multicomponent exercise that included aerobic and resistance training (S. Colcombe & Kramer, 2003). On the other hand, resistance training has been shown to improve comorbidities associated with cognitive impairment in diabetes, such as hypertension, dyslipidemia, and glycemic control (Helzner et al., 2009; Zhao et al., 2018). Although the American College of Sports Medicine recommends including resistance exercises in the daily regimen for individuals with diabetes, the effects of resistance exercise on executive function remain inconsistent in the present study (Kanaley et al., 2022). Some studies suggest that aerobic exercise may be more effective than resistance exercise in managing blood glucose levels in type 2 diabetes mellitus patients, a factor known to be linked to improved executive function (Benham et al., 2020; Helzner et al., 2009; Mannucci et al., 2021; Nery et al., 2017; Zhao et al., 2018). Most of the studies in this analysis involved type 2 diabetes mellitus patients. Given that better glucose control is associated with improved executive function, it is plausible that resistance exercise may have inherent limitations in enhancing executive function (Schwartz et al., 2024; Sugimoto et al., 2023; S. Zhang et al., 2023). Additionally, the lack of more clinical, physiological, biochemical, and epidemiological evidence, coupled with the variability in exercise plans, may also limit the ability of this study to fully uncover the potential role of multicomponent and resistance exercises. Furthermore, Wang H et al. examined the effects of three acute exercise interventions (aerobic, resistance, and multicomponent exercise) on the executive function of hospitalized patients with type 2 diabetes mellitus, and the results showed that each type of exercise activated the brain regions associated with executive function (H. Wang et al., 2023). Nevertheless, due to the small sample size (n = 30) and the short intervention duration (acute), the clinical significance of these findings requires further verified.

There are no previous studies that report on the dose–response relationship of exercise between exercise and executive function in diabetes. The subgroup results tentatively suggest that exercise lasting at least 45 min per session and for 24 weeks or more might hold potential for influencing executive function in individuals with diabetes. However, these findings remain strictly hypothesis-generating. Similar to our results, exercise intervention patterns lasting 13–26 weeks and 45–60 min per week were associated with improvements in executive function in healthy older adults, suggesting that longer interventions may be necessary to alleviate age-related decline in executive function (Xiong et al., 2021; M. Zhang et al., 2023). Diabetes and aging are known to have synergistic pathogenic effects (Z. Li et al., 2022). Given that diabetes and aging have synergistic pathogenic effects, the elderly diabetic population in this study may explain the need for longer exercise interventions compared to healthy individuals (as shown in Supplement Table S1). Interestingly, we found that 120 min of total exercise per week did not significantly impact executive function. While a meta-analysis of exercise interventions for older adults with mild cognitive impairment showed that moderate-frequency exercise (3–4 times per week) was more effective than low-frequency exercise (1–2 times per week), most studies in this meta-analysis used a frequency of 3 times per week (Lin et al., 2022). However, current research included in this meta-analysis generally used exercise frequency of 3 times per week, which may reflect a balance between patient adherence and safety. This restriction on exercise frequency may prevent us from investigating the effect of total exercise time per week after clarifying the optimal duration of a single exercise session. Future research should further explore the impact of different exercise frequencies on cognitive outcomes. Although the overall pooled effect on executive function was non-significant, a sensitivity analysis restricted to trials that prespecified executive function as a primary outcome revealed a significant benefit, and outcome status significantly moderated the effect. This suggests that the null overall result was largely driven by heterogeneous secondary-outcome studies, in which executive function was measured less rigorously and may be more susceptible to selective reporting. Adequately powered trials prespecifying executive function as a primary outcome are warranted to confirm this benefit.

### 4.2. Memory

As reported in most randomized controlled trials and systematic review studies, this meta-analysis found that exercise significantly improved memory in diabetic patients (Baker et al., 2010; Devore et al., 2009; Dyer et al., 2020; Espeland et al., 2017; Z. Sun et al., 2024). Subgroup analysis revealed that both multicomponent and aerobic exercises provided significant benefits for memory, with multicomponent exercise showing particularly strong effects. Callisaya ML and Espeland MA et al. conducted 2 clinical randomized controlled trials of multimodal exercise, demonstrating that long-term multicomponent exercise significantly delayed or improved global cognitive and memory function in patients with type 2 diabetes mellitus (Callisaya et al., 2017; Espeland et al., 2017). This may be partly attributed to the fact that multicomponent exercise typically requires participants to perform a series of different exercise programs successively. In order to remember and complete these exercise programs, participants presented additional memory challenges and yielded greater memory enhancements. Meanwhile, aerobic exercise has been shown to enhance memory by increasing brain volume, enhancing neuroplasticity and enhancing the release of brain-derived neurotrophic factors, which ultimately improve cognitive dysfunction in diabetic patients (S. J. Colcombe et al., 2006; Sáez de Asteasu et al., 2019; Vaughan et al., 2014; J. Zhang et al., 2024). Interestingly, despite its richer action setting, resistance exercise did not show significant improvements in memory. Only two studies examining the effects of resistance training on memory were included, which may have led to insufficient statistical power to effectively capture its potential effects. Furthermore, the differential impact of various exercise types on memory in diabetes remains unclear, and there is a lack of research on the interaction between different exercise modalities when combined. Based on the current evidence, both multicomponent and aerobic exercises are recommended as the primary strategies for improving memory in diabetic patients. However, the potential benefits of resistance exercise require further validation through higher-quality studies.

To our knowledge, there is a lack of studies investigating the dose–response relationship of exercise in improving memory in diabetes. Our analysis indicates that exercise training lasting less than 60 min per session and totaling less than 120 min per week for 12 weeks or more can significantly improve memory in diabetic patients. In an epidemiologic study, 1550 people with diabetes were followed for nearly two years, revealing that long-term, higher levels of physical activity were associated with enhanced memory, even after adjusting for age and education (Devore et al., 2009). Similarly, Watson GS et al. found that 180 min of walking or jogging per week for 24–48 weeks, combined with dietary modifications, significantly improved verbal memory in Japanese-Americans with impaired glucose tolerance. This aligns with our findings, though dietary modifications in their study may have contributed to some of the observed heterogeneity (Watson et al., 2006). However, due to the lack of consistent reporting on exercise intensity across studies, we were unable to determine its specific impact on memory (Furlano et al., 2023; Liu et al., 2024; Silveira-Rodrigues et al., 2021; Suh et al., 2019; Zhao et al., 2022). It is known that higher exercise intensity can delay age-related memory decline in healthy individuals (M. Zhang et al., 2023). Therefore, more comprehensive and uniform trials are needed in the future to assess the dose–response effects of exercise parameters on memory benefits.

### 4.3. Attention

Although exercise intervention demonstrated significant improvement in various cognitive domains (such as executive function, working memory and global cognition), our subgroup analysis revealed no significant effect on attention in individuals with diabetes. Two previous systematic reviews have reported that exercise can improve attention in diabetes (Monette et al., 2014; Pelimanni & Jehkonen, 2019). However, recent reviews of non-pharmacological interventions in diabetes have pointed to a lack of statistical quantification of clinical evidence, suggesting the need for further investigation (Cooke et al., 2020; Dyer et al., 2020; Zhao et al., 2018). In fact, exercise intervention positively changes the attention domain of diabetic patients to a certain extent, but it may be affected by the length of the intervention period. For instance, older individuals with diabetes showed significant improvements in attention after 48 weeks of progressive resistance training (Zhao et al., 2022). In contrast, a single 24-week clinical intervention involving aerobic walking did not affect attention in older adults with diabetes (Shellington et al., 2018). These findings suggest that the effectiveness of exercise on attention may be enhanced by longer intervention periods.

Interestingly, some studies found that multicomponent exercise, even with a shorter intervention period (e.g., 8 weeks), can significantly improve attention and concentration in middle-aged and elderly patients with type 2 diabetes mellitus (Fiocco et al., 2013; Silveira-Rodrigues et al., 2021). It is known that multicomponent exercise usually integrates various forms of exercise such as aerobic exercise, resistance training, flexibility training, and balance training (Forsyth et al., 2024; L. Li et al., 2021; Z. Sun et al., 2024; Yan et al., 2024). The attention-related brain regions of the subjects (such as the prefrontal lobe and subthalamic nucleus, etc.) may be continuously stimulated under this diverse movement pattern, thereby being able to obtain exercise benefits in a shorter period (Soh et al., 2024). It is worth noting that of the 4 studies included in this study, the remaining 3 used either aerobic exercise combined with resistance exercise or other forms of physical training (Ghodrati et al., 2023; Shellington et al., 2018; Silveira-Rodrigues et al., 2021). In contrast, the randomized controlled trials by Chen Y and colleagues found that tai chi, compared with aerobic walking, significantly improved attention in patients with type 2 diabetes mellitus and mild cognitive impairment (Y. Chen et al., 2023). Tai Chi, a mind-body exercise characterized by a high degree of coordination between thought, breath, and movement, may stimulate synaptic development and improve attention (Wayne et al., 2014; Wei et al., 2022). However, due to the small sample size, this study did not fully reveal the differential effects of different exercise types on attention, so its results should be interpreted cautiously. In the future, more randomized controlled trials with a standardized design based on the FITT (frequency, intensity, time and type) principle are needed to investigate the effects of exercise on attention.

### 4.4. Global Cognition

This meta-analysis examined the impact of exercise on global cognition in individuals with diabetes. Subgroup analysis revealed that both aerobic and multicomponent exercise significantly improved global cognition, with both exercise durations (under or over 45 min per session) and intervention periods (less than or more than 24 weeks) showing positive effects. The above results are also similar to the results of several previous systematic reviews and meta-analyses, suggesting that long-term exercise provides a broad dose–response benefit for global cognition in diabetic patients (Cooke et al., 2020; Lu et al., 2024; R. Wang et al., 2021). Long-term regular exercise is known to offer multiple metabolic benefits in people with diabetes. For instance, exercise increases activity and blood flow in brain regions by enhancing insulin sensitivity (Olver et al., 2019). In addition, exercise intervention effectively reduced proinflammatory cytokines such as TNF-α, IFN-γ, and IL-1β in middle-aged and elderly patients with type 2 diabetes mellitus, which was associated with improvement in cognitive impairment (Baek et al., 2022). Thus, rather than the specific mechanisms of improvement in previous cognitive domains, the improvement in global cognition may be more dependent on the accumulation of systemic health benefits induced by exercise.

### 4.5. Motor Performance as a Correlate of Cognitive Benefits

This study demonstrates that exercise interventions significantly enhance motor performance in diabetic individuals, with multicomponent exercise and short-term interventions (<12 weeks) the most significant. Improvements in motor function (reflected in outcomes such as walking endurance) may indicate not only enhanced physical capacity, but also provide indirect evidence for neurobehavioral cognitive benefits (Debarnot et al., 2014; Latino & Tafuri, 2024; Sakai et al., 2023). Crucially, most studies assessed motor performance via the 6-min walk test, a measure requiring integrated prefrontal–cerebellar–basal ganglia network activation that concurrently underpins executive function (Furlano et al., 2023; Ghahfarrokhi et al., 2024; Plotnikoff et al., 2010; Ploydang et al., 2023). The 6-min walk test necessitates sustained sensorimotor integration and cognitive control, engaging prefrontal executive networks (Hayashi et al., 2018; Hayes et al., 2014; Khalil, 2025). Increased 6-min walk test distance correlates with elevated brain-derived neurotrophic factors, which promotes hippocampal synaptic plasticity and enhances executive and memory functions. Subgroup analyses revealed significantly improved motor performance within <12 weeks, whereas memory and executive functions required ≥12 and ≥24 weeks, respectively. This temporal hierarchy suggests that motor performance may serve as an early biomarker for cognitive intervention efficacy in diabetes. However, further clinical validation is necessary.

### 4.6. Limitations

This study adhered to the guidelines outlined in the PRISMA statement. However, there are 4 limitations that should be addressed in future research. (1) Although the literature search followed the PRISMA criteria rigorously, unpublished works may have been overlooked. (2) The conclusions drawn for each cognitive subdomain must be interpreted through the lens of their different statistical heterogeneity values. While the lack of heterogeneity in global cognition indicates a robust and uniform treatment effect across diverse cohorts, the moderate-to-high heterogeneity in executive function and memory reflects substantial clinical and methodological divergence. This statistical variation indicates that the efficacy of exercise in these specific domains is highly sensitive to protocol differences, meaning that our overall pooled estimates should be viewed with caution and that the respective subgroup findings remain strictly exploratory. This heterogeneity is likely driven by the clinical diversity of the enrolled cohorts and variability in exercise prescription parameters (e.g., training strategy and equipment), which might not be uniformly standardized across trials. Additionally, exercise intensity, a core component of any prescription, was inconsistently reported and could not be analyzed as a moderator, so studies within the same subgroup may have differed substantially in intensity, introducing unmeasured heterogeneity that could confound the dose–response patterns and leaving the suggested exercise parameters incomplete. Future trials should report exercise intensity and weekly frequency using standardized metrics (e.g., %HRmax, %VO_2_peak, RPE, or %1RM) to enable intensity-stratified, dose–response analyses. (3) The limited number of trials investigating exercise interventions in diabetes, combined with variations in participant characteristics (e.g., disease type, age, gender, and BMI), may compromise the credibility of the results. (4) The absence of multidimensional cognitive indicators represents another limitation. While multiple cognitive assessment tools exist, significant differences in measurement methods and reporting formats were observed across studies. At the same time, the lack of neuroimaging and molecular biological data may reduce the reliability of clinical conclusions.

## 5. Conclusions

Our systematic review and meta-analysis support the beneficial effects of exercise on global cognition and memory in patients with diabetes. For memory enhancement, aerobic or multicomponent exercise protocols of ≥12 weeks, ≤120 min of total weekly exercise, and ≤60 min per session are more recommended. Although the overall pooled effect on executive function did not reach statistical significance, exploratory subgroup findings point toward potential domain-specific benefits under targeted exercise parameters (such as aerobic exercise of longer duration). These dose–response patterns should therefore be viewed as exploratory and hypothesis-generating, warranting confirmation in adequately powered, head-to-head trials before specific exercise prescriptions can be recommended.

## Figures and Tables

**Table 1:** Study inclusion criteria based on the PICOS model.

Parameter	Inclusion Criteria
Population	Diagnosed with diabetes mellitus
Intervention	Physical exercise therapy
Comparator	No intervention
Outcome	Cognitive function and its subdomains (Supplementary Table S2)
Study design	Randomized control trials

**Table 2:** Subgroup analysis of the effects of different moderating variables on executive function.

Moderator Variable	Stratified Subgroup	Numbers Included in the Study	SMD (95%CI)	*p* Score	*I*^2^ (%)
Exercise type	Multicomponent exercise	3	0.31 [−0.39, 1.02]	0.3851	72.5
	Resistance exercise	3	−0.11 [−0.96, 0.74]	0.7986	77.6
	Aerobic exercise	4	−0.30 [−0.50, −0.10]	0.0039	0
Exercise duration (minutes)	≥45	5	−0.40 [−0.80, −0.01]	0.0463	45.7
	<45	5	0.13 [−0.28, 0.53]	0.5368	68.2
Total exercise time (minutes/per week)	>120	6	−0.26 [−0.70, 0.17]	0.2386	62.6
	≤120	4	0.06 [−0.38, 0.51]	0.7784	66.1
Exercise period (weeks)	≥24	4	−0.27 [−0.52, −0.01]	0.0383	48.7
	<24	6	0.13 [−0.35, 0.60]	0.6042	63

**Table 3:** Subgroup analysis of the effects of different moderating variables on memory.

Moderator Variable	Stratified Subgroup	Numbers Included in the Study	SMD (95%CI)	*p* Score	*I*^2^ (%)
Exercise type	Multicomponent exercise	3	0.45 [0.06, 0.83]	0.0224	13.1
	Resistance exercise	2	0.20 [−0.15, 0.56]	0.2678	86.3
	Aerobic exercise	3	0.27 [0.06, 0.48]	0.0131	25
Exercise duration (minutes)	>60	2	0.34 [−0.11, 0.80]	0.1415	0
	≤60	6	0.28 [0.10, 0.45]	0.002	58.6
Total exercise time (minutes/per week)	>120	5	0.19 [−0.07, 0.45]	0.1551	55.7
	≤120	3	0.35 [0.14, 0.56]	0.0012	35.3
Exercise period (weeks)	≥12	6	0.32 [0.15, 0.49]	0.0003	51.5
	<12	2	−0.00 [−0.50, 0.49]	0.9863	0

**Table 4:** Subgroup analysis of the effects of different moderating variables on attention.

Moderator Variable	Stratified Subgroup	Numbers Included in the Study	SMD (95%CI)	*p* Score	*I*^2^ (%)
Exercise type	Multicomponent exercise	2	−0.28 [−0.84, 0.27]	0.3160	34.8
	Aerobic exercise	2	0.19 [−0.03, 0.41]	0.0874	0
Exercise duration (minutes)	≥45	2	−0.40 [−0.99, 0.19]	0.1853	0
	<45	2	0.20 [−0.02, 0.42]	0.0739	0
Total exercise time (minutes/per week)	>120	2	−0.40 [−0.99, 0.19]	0.1853	0
	≤120	2	0.20 [−0.03, 0.42]	0.0739	0
Exercise period (weeks)	≥24	2	0.19 [−0.03, 0.41]	0.0874	0
	<24	2	−0.28 [−0.84, 0.27]	0.3160	34.8

**Table 5:** Subgroup analysis of the effects of different moderating variables on global cognition.

Moderator Variable	Stratified Subgroup	Numbers Included in the Study	SMD (95%CI)	*p* Score	*I*^2^ (%)
Exercise type	Multicomponent exercise	2	0.55 [0.05, 1.06]	0.0316	0
	Aerobic exercise	4	0.47 [0.29, 0.66]	<0.0001	24
Exercise duration (minutes)	≥45	4	0.43 [0.15, 0.71]	0.0029	19.6
	<45	3	0.52 [0.31, 0.72]	<0.0001	0
Total exercise time (minutes/per week)	>120	4	0.43 [0.15, 0.71]	0.0029	19.6
	≤120	3	0.52 [0.31, 0.72]	<0.0001	0
Exercise period (weeks)	≥24	3	0.50 [0.30, 0.69]	<0.0001	0
	<24	4	0.45 [0.14, 0.77]	0.0050	9.3

## Data Availability

No new data were created or analyzed in this study. Data sharing is not applicable to this article.

## Supplementary Materials

The following supporting information can be downloaded at: https://www.mdpi.com/article/10.3390/bs16071218/s1. Table S1. The details of cognitive assessment tools of included studies, Table S2. The measurement tools for different outcomes, Table S3. Methodological quality assessment of included studies, Table S4. Search strategy; Table S5: PRISMA 2020 Checklist; Figure S1. Forest plot of physical exercise in executive function, type of physical exercise, Figure S2. Forest plot of physical exercise in executive function, duration of exercise, Figure S3. Forest plot of physical exercise in executive function, total exercise time per week, Figure S4. Forest plot of physical exercise in executive function, period of physical exercise, Figure S5. Forest plot of physical exercise in memory, type of physical exercise, Figure S6. Forest plot of physical exercise in memory, duration of exercise, Figure S7. Forest plot of physical exercise in memory, total exercise time per week, Figure S8. Forest plot of physical exercise in memory, period of physical exercise, Figure S9. Forest plot of physical exercise in attention, Figure S10. Forest plot of physical exercise in attention, duration of exercise, Figure S11. Forest plot of physical exercise in attention, total exercise time per week, Figure S12. Forest plot of physical exercise in attention, period of physical exercise, Figure S13. Forest plot of physical exercise in global cognition, type of physical exercise, Figure S14. Forest plot of physical exercise in global cognition, duration of exercise, Figure S15. Forest plot of physical exercise in global cognition, total exercise time per week, Figure S16. Forest plot of physical exercise in global cognition, period of physical exercise, Figure S17. Meta-regression analyses of exercise variables and cognitive subdomains, Figure S18. Meta-regression analyses of exercise variables and cognitive subdomains, Figure S19. Meta-regression analyses of exercise variables and cognitive subdomains, Figure S20. Forest plot of physical exercise in motor performance, Figure S21. Forest plot of physical exercise in global cognition, type of physical exercise, Figure S22. Forest plot of physical exercise in global cognition, period of physical exercise; Figure S23. Effect of exercise on executive function stratified by outcome status (primary vs. secondary cognitive outcome), Figure S24. Sensitivity analysis of the effect of exercise on executive function, restricted to studies in which executive function was a prespecified primary outcome.
