# Supplementary material for: Optimizing Exercise Prescriptions for Cognitive Subdomains in Diabetes: A Systematic Review and Meta-Analysis of Dose–Response Variables
Source: Behav Sci (Basel). 2026 Jul 18;16(7):1218. doi: 10.3390/bs16071218 (PMC13405410; doi:10.3390/bs16071218)
Supplement: Supplementary file 1 [file behavsci-16-01218-s001.zip › behavsci-4348082-supplementary.pdf]

Figure S1. Forest plot of physical exercise in executive function, type of physical exercise; 95%CI = 95% confidence interval; I^2 = inconsistency between studies; SMD = standardized mean difference.

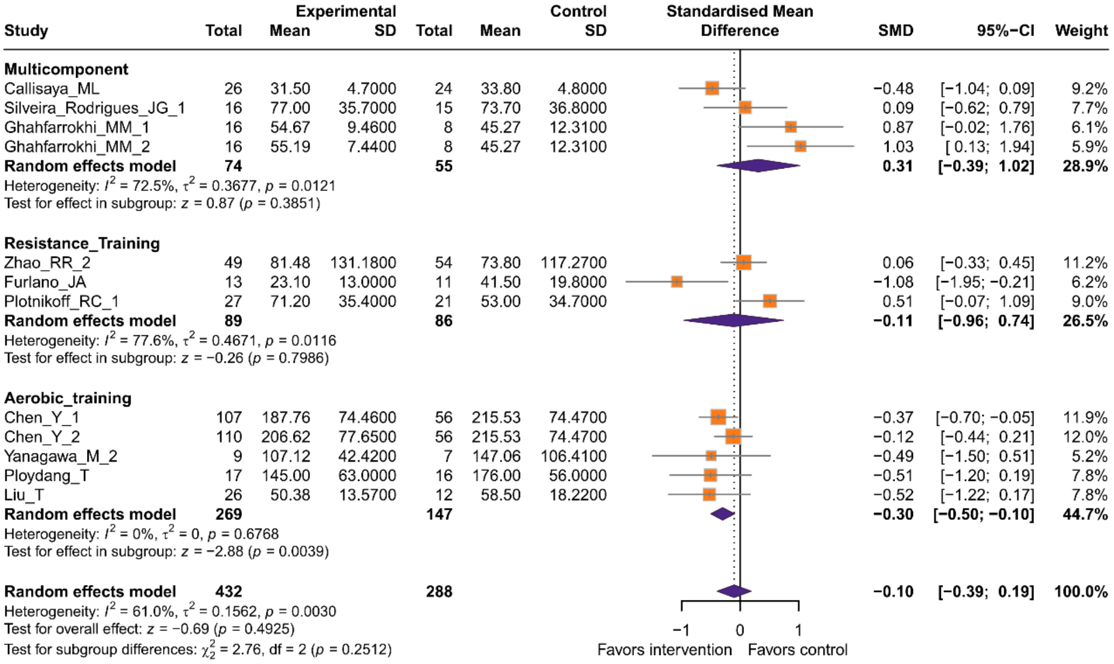

Figure S2. Forest plot of physical exercise in executive function, duration of exercise; 95%CI = 95% confidence interval; I^2 = inconsistency between studies; SMD = standardized mean difference.

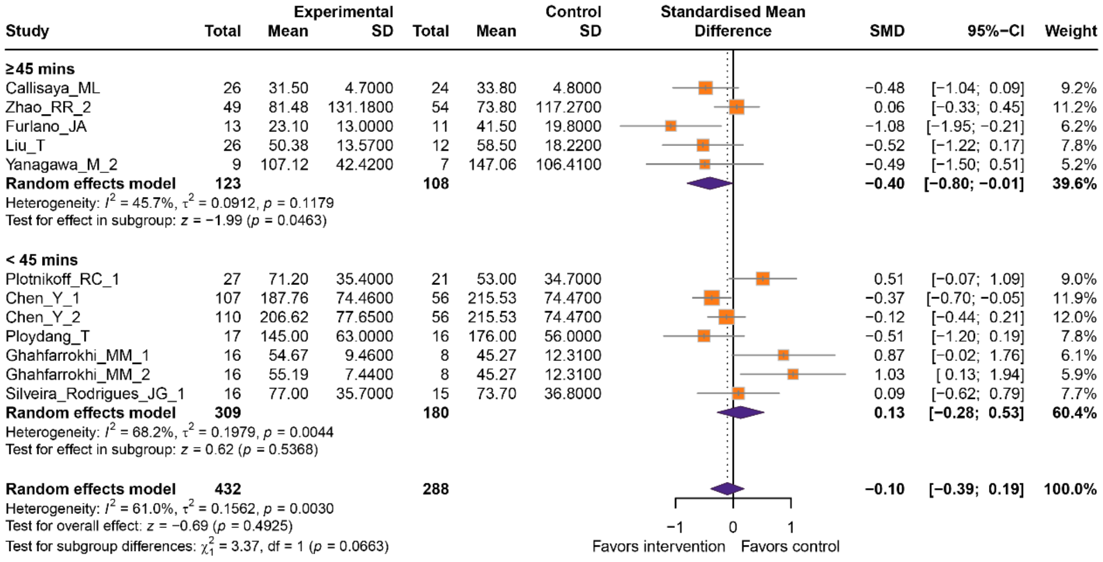

Figure S3. Forest plot of physical exercise in executive function, total exercise time per week; 95%CI = 95% confidence interval; I^2 = inconsistency between studies; SMD = standardized mean difference.

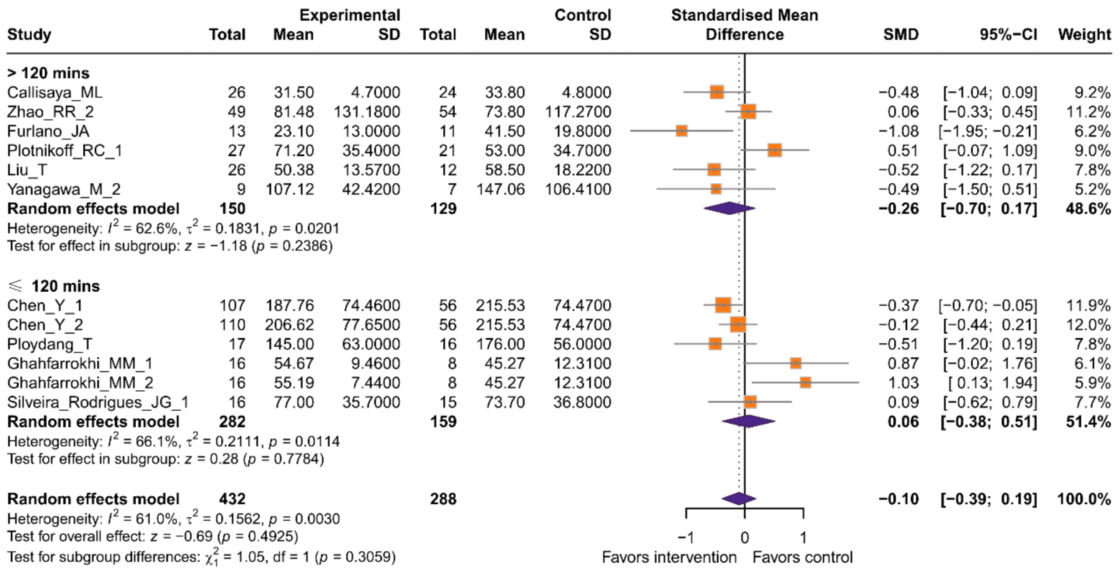

Figure S4. Forest plot of physical exercise in executive function, period of physical exercise; 95%CI = 95% confidence interval; I^2 = inconsistency between studies; SMD = standardized mean difference.

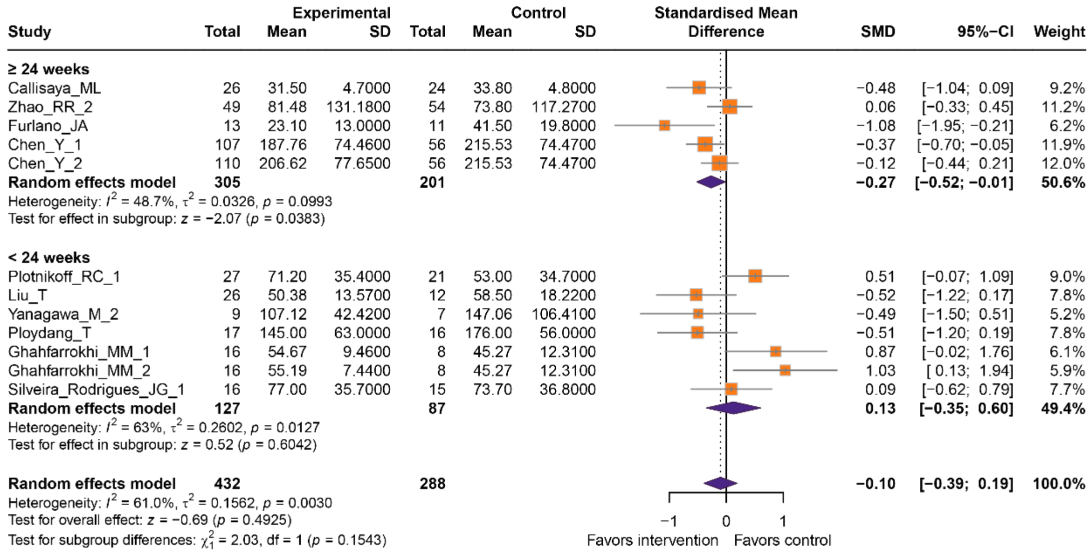

Figure S5. Forest plot of physical exercise in memory, type of physical exercise; 95%CI = 95% confidence interval; df = degree of freedom; I^2 = inconsistency between studies; SMD = standardized mean difference.

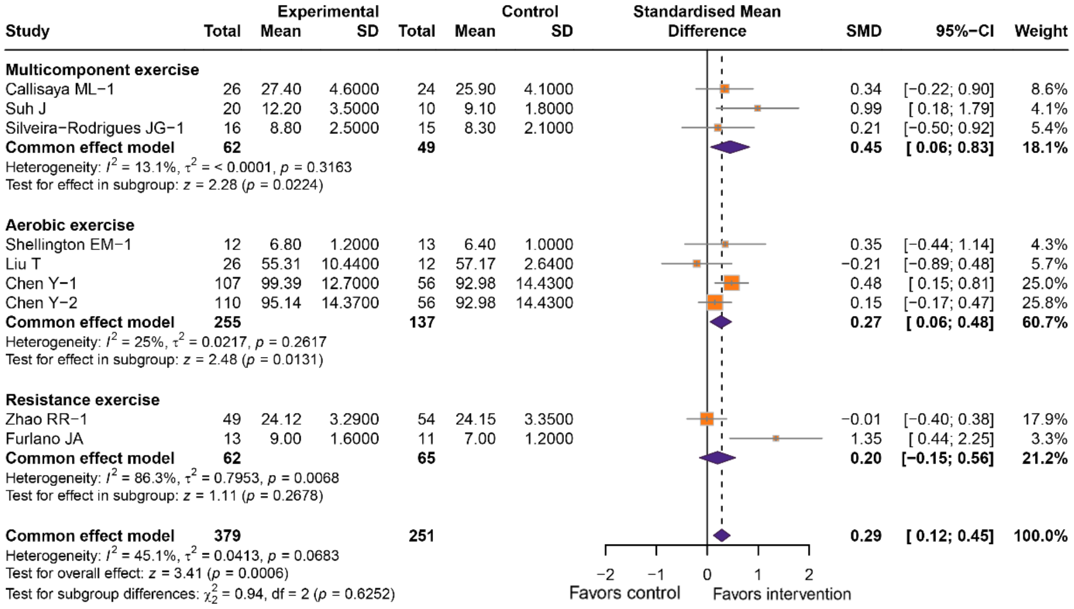

Figure S6. Forest plot of physical exercise in memory, duration of exercise; 95%CI = 95% confidence interval; *df* = degree of freedom; I^2 = inconsistency between studies; SMD = standardized mean difference.

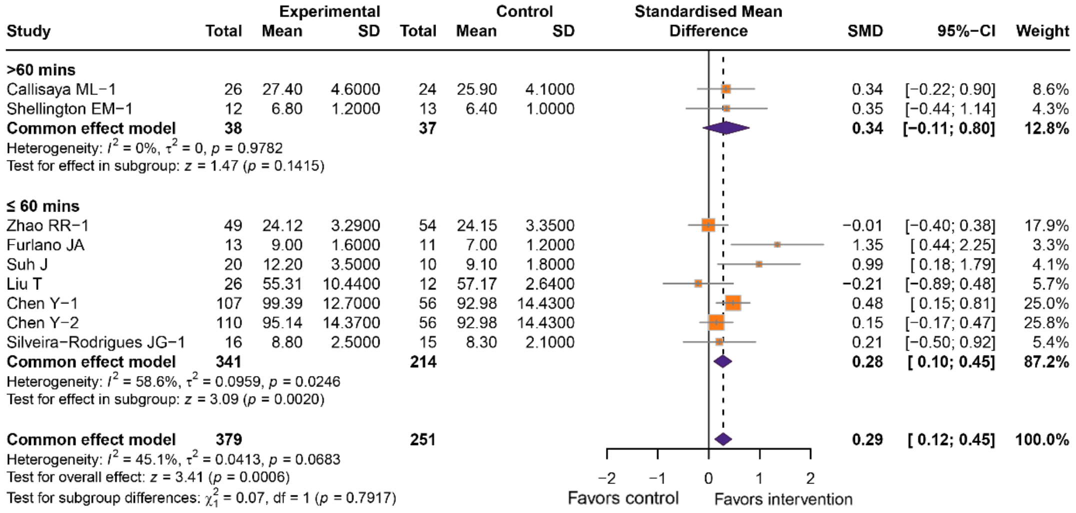

Figure S7. Forest plot of physical exercise in memory, total exercise time per week; 95%CI = 95% confidence interval; *df* = degree of freedom; I^2 = inconsistency between studies; SMD = standardized mean difference.

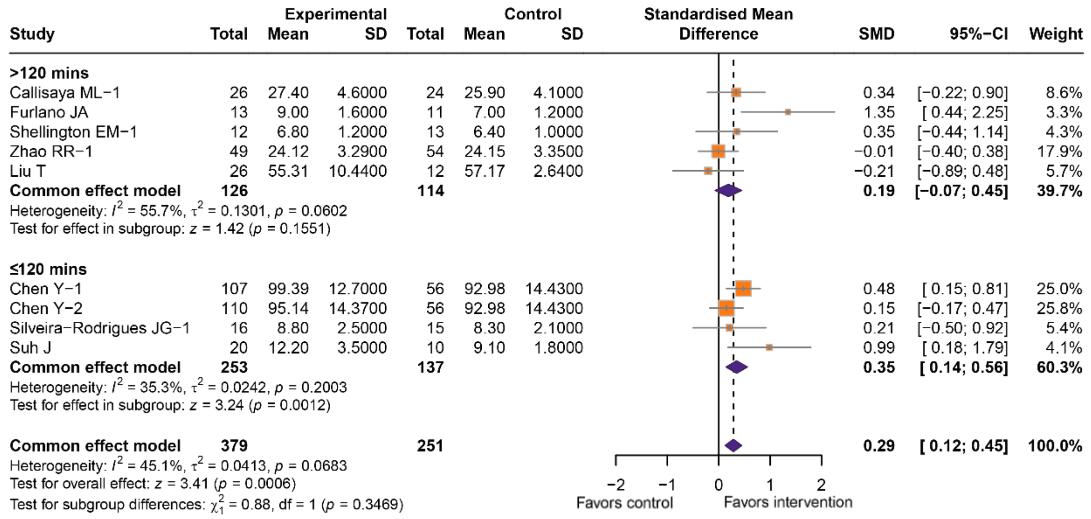

Figure S8. Forest plot of physical exercise in memory, period of physical exercise; 95%CI = 95% confidence interval; *df* = degree of freedom; I^2 = inconsistency between studies; SMD = standardized mean difference.

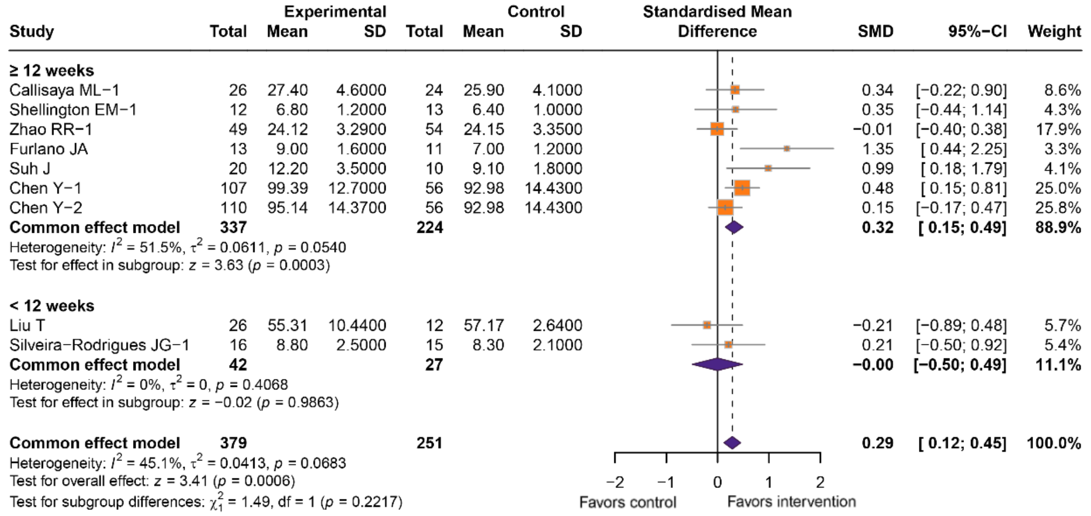

Figure S9. Forest plot of physical exercise in attention; 95%CI = 95% confidence interval, type of physical exercise; *df* = degree of freedom; I^2 = inconsistency between studies; SMD = standardized mean difference.

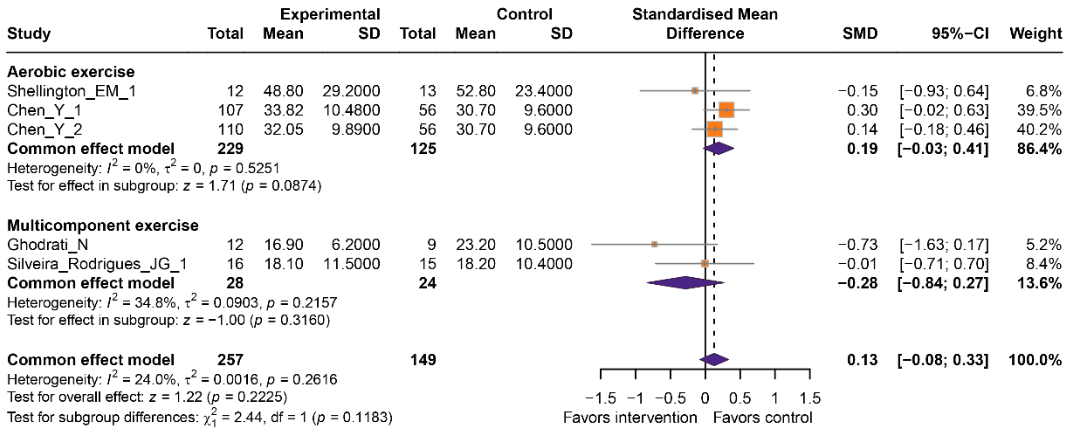

Figure S10. Forest plot of physical exercise in attention, duration of exercise; 95%CI = 95% confidence interval; *df* = degree of freedom; I^2 = inconsistency between studies; SMD = standardized mean difference.

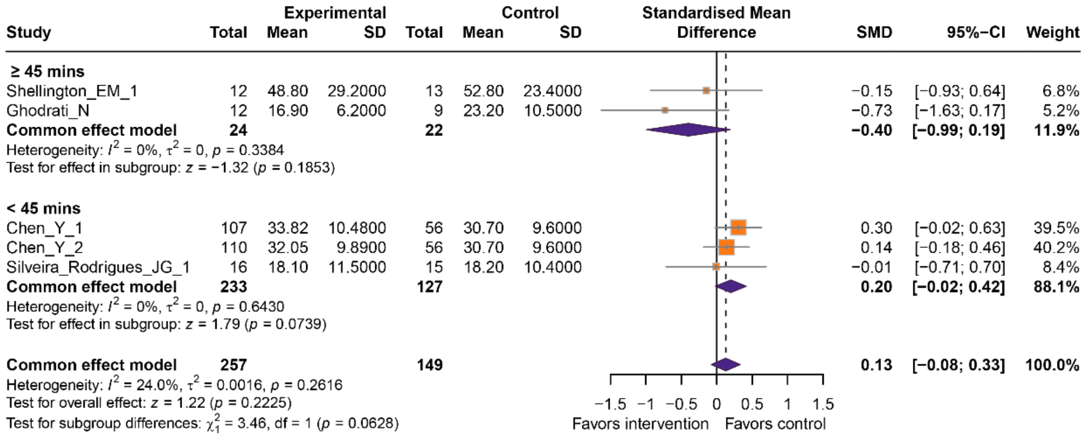

Figure S11. Forest plot of physical exercise in attention, total exercise time per week; 95%CI = 95% confidence interval; *df* = degree of freedom; I^2 = inconsistency between studies; SMD = standardized mean difference.

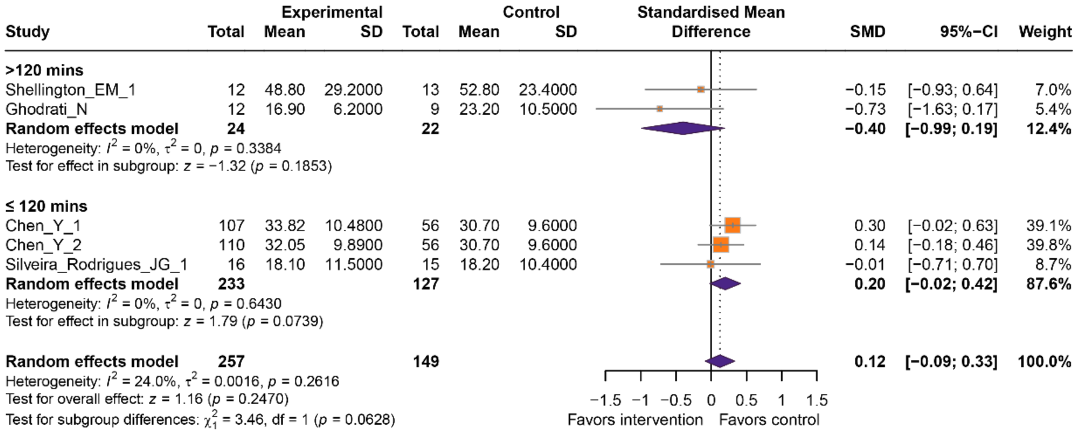

Figure S12. Forest plot of physical exercise in attention, period of physical exercise; 95%CI = 95% confidence interval; *df* = degree of freedom; I^2 = inconsistency between studies; SMD = standardized mean difference.

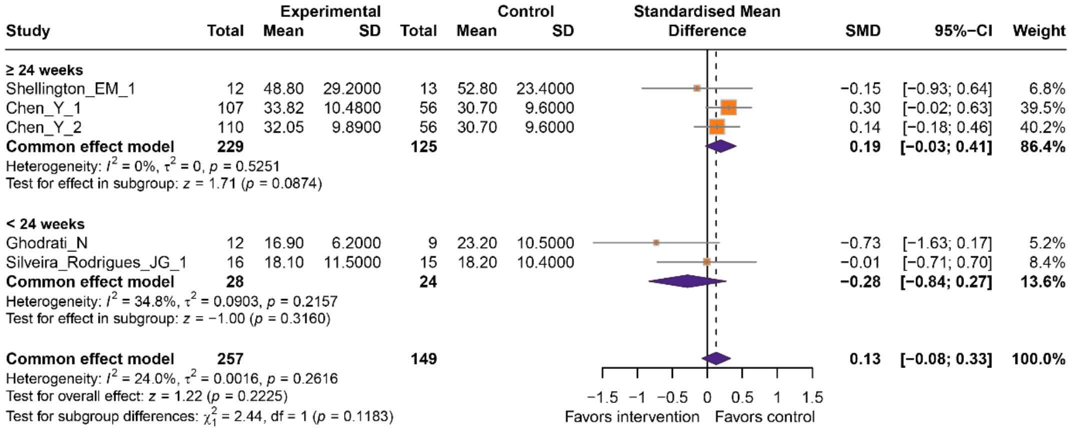

Figure S13. Forest plot of physical exercise in global cognition, type of physical exercise; 95%CI = 95% confidence interval; *df* = degree of freedom; I^2 = inconsistency between studies; SMD = standardized mean difference.

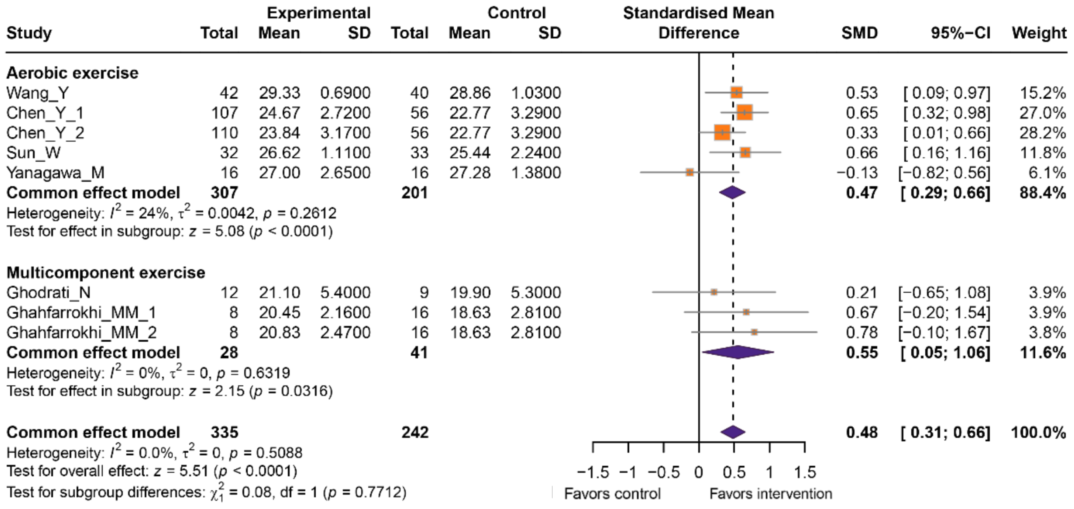

Figure S14. Forest plot of physical exercise in global cognition, duration of exercise; 95%CI = 95% confidence interval; *df* = degree of freedom; I^2 = inconsistency between studies; SMD = standardized mean difference.

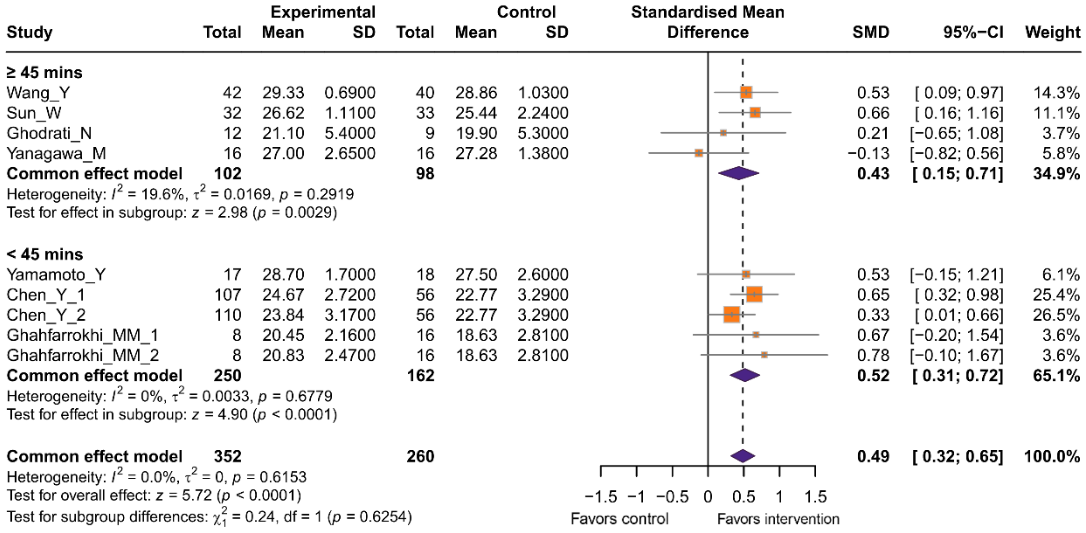

Figure S15. Forest plot of physical exercise in global cognition, total exercise time per week; 95%CI = 95% confidence interval; *df* = degree of freedom; I^2 = inconsistency between studies; SMD = standardized mean difference.

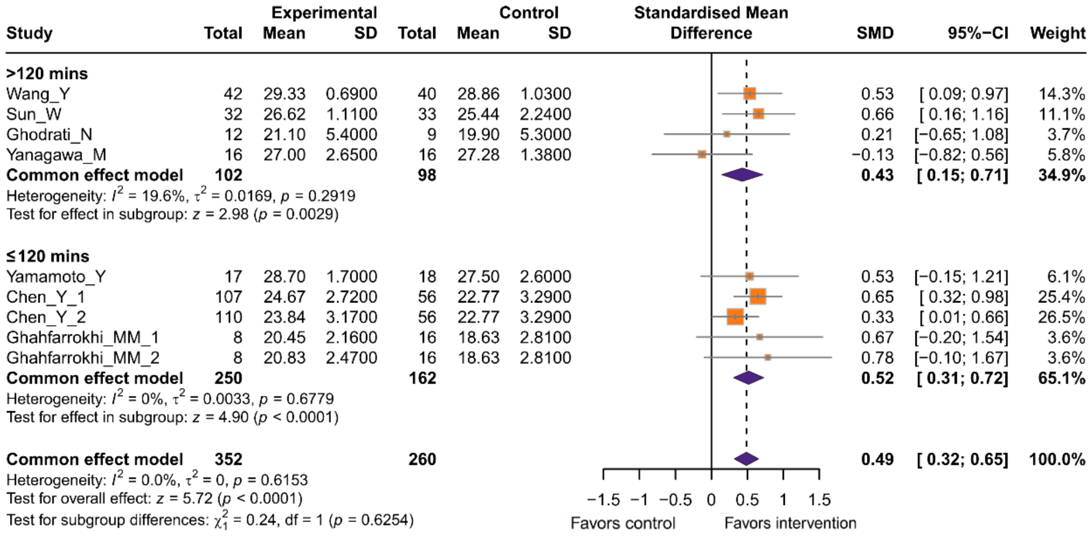

Figure S16. Forest plot of physical exercise in global cognition, period of physical exercise; 95%CI = 95% confidence interval; *df* = degree of freedom; I^2 = inconsistency between studies; SMD = standardized mean difference.

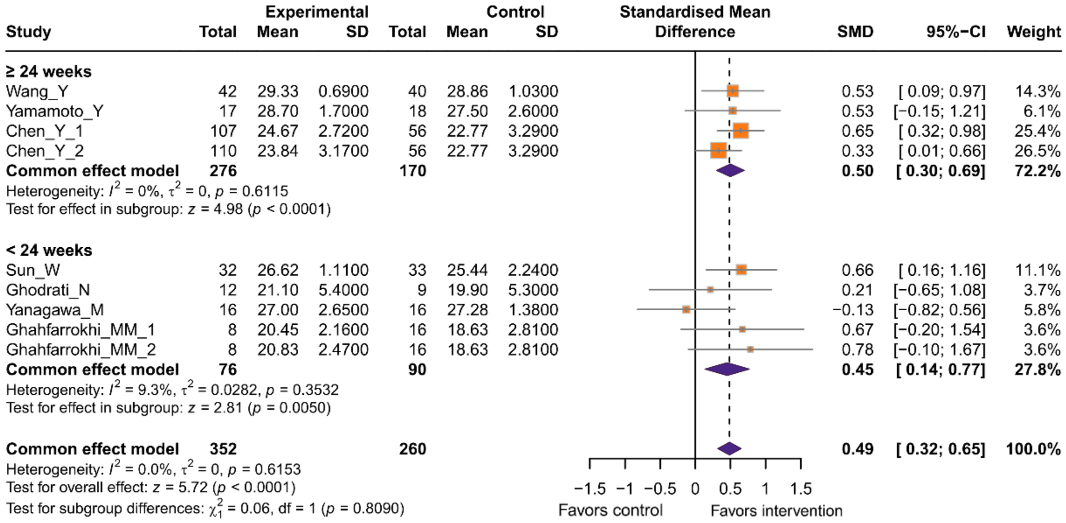

Figure S17. Meta-regression analyses of exercise variables and cognitive subdomains. A. Meta-regression analysis of intervention period and memory; B. Meta-regression analysis of single-session exercise duration and memory; C. Meta-regression analysis of total weekly exercise duration and memory.

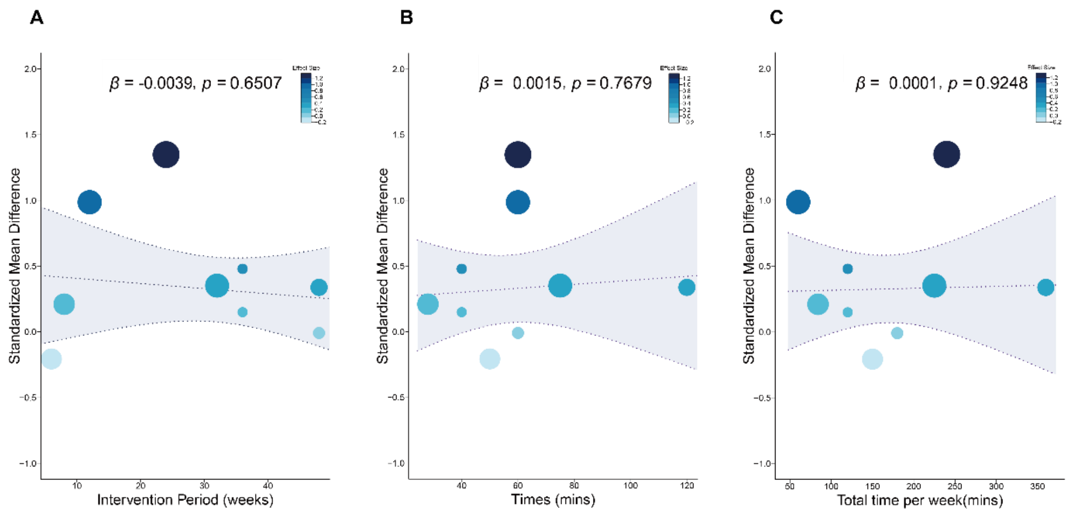

Figure S18. Meta-regression analyses of exercise variables and cognitive subdomains. A. Meta-regression analysis of intervention period and attention; B. Meta-regression analysis of single-session exercise duration and attention; C. Meta-regression analysis of total weekly exercise duration and attention.

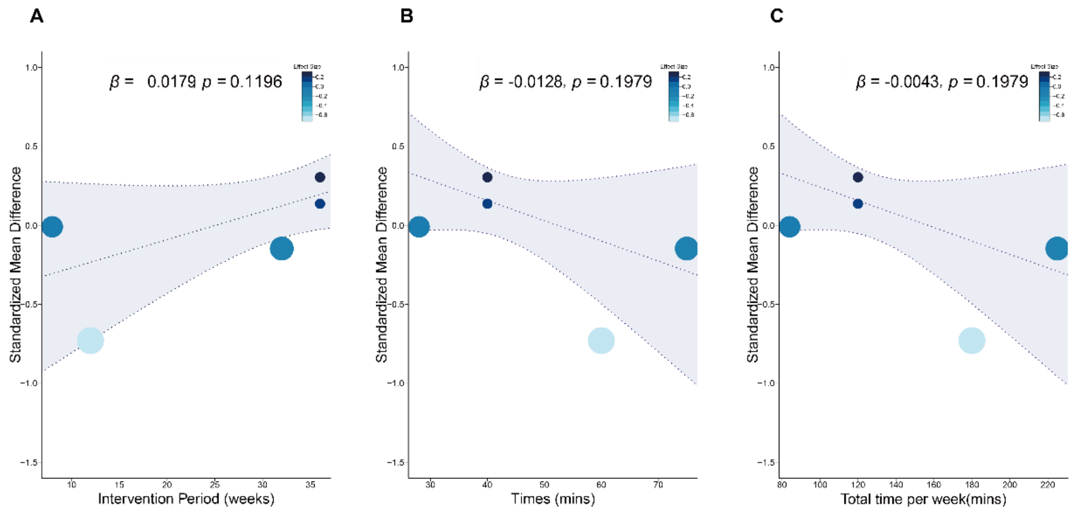

Figure S19. Meta-regression analyses of exercise variables and cognitive subdomains. A. Meta-regression analysis of intervention period and global cognition; B. Meta-regression analysis of single-session exercise duration and global cognition; C. Meta-regression analysis of total weekly exercise duration and global cognition.

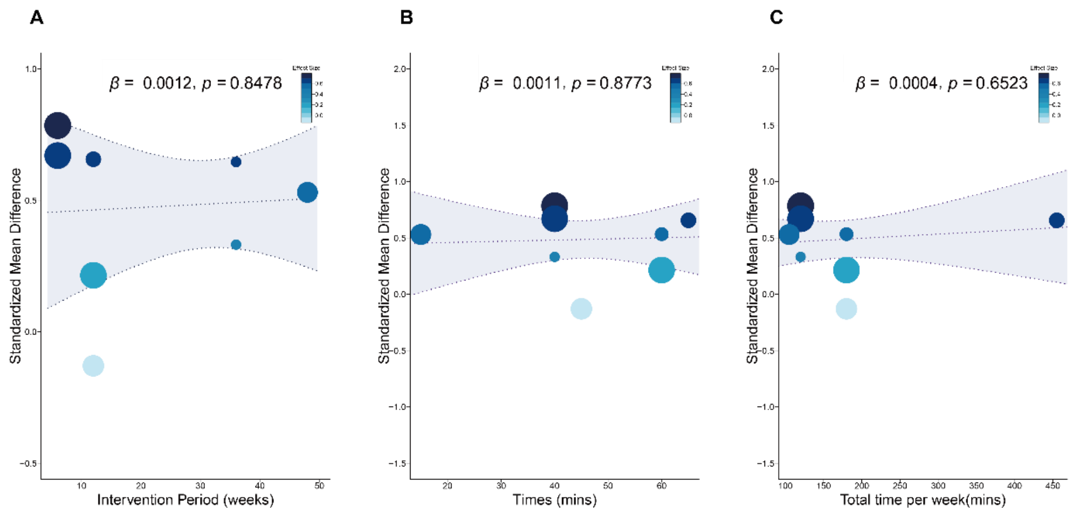

Figure S20. Forest plot of physical exercise in motor performance; 95%CI = 95% confidence interval; *df* = degree of freedom; I^2 = inconsistency between studies; SMD = standardized mean difference.

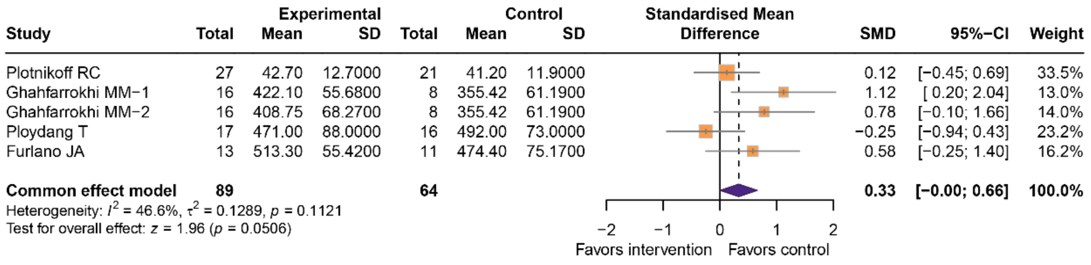

Figure S21. Forest plot of physical exercise in global cognition, type of physical exercise; 95%CI = 95% confidence interval; *df* = degree of freedom; I^2 = inconsistency between studies; SMD = standardized mean difference.

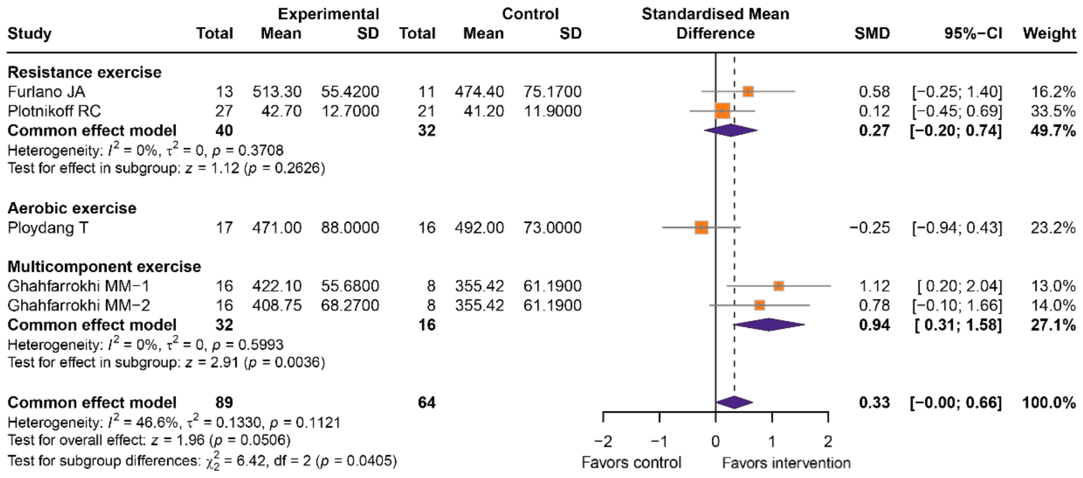

Figure S22. Forest plot of physical exercise in global cognition, period

of physical exercise; 95%CI = 95% confidence interval; *df* = degree of freedom; I^2 = inconsistency between studies; SMD = standardized mean difference.

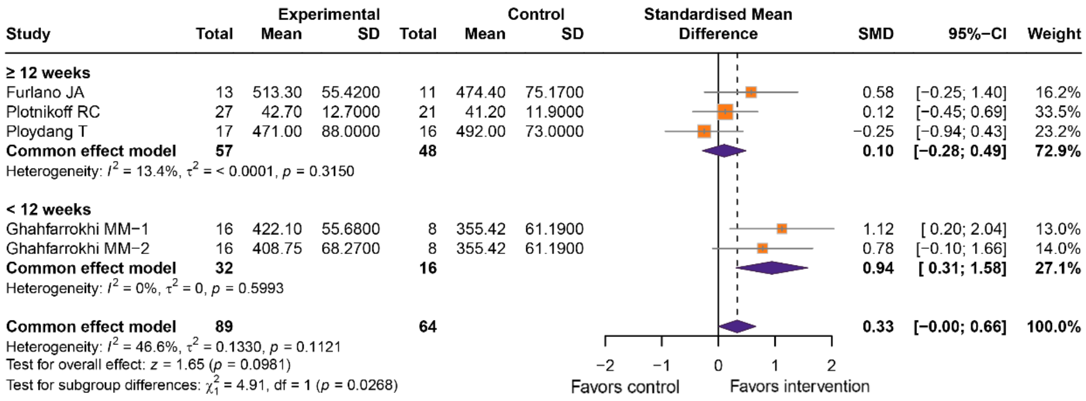

Figure S23. Effect of exercise on executive function stratified by outcome status (primary vs. secondary cognitive outcome); 95%CI = 95% confidence interval; *df* = degree of freedom; I^2 = inconsistency between studies; SMD = standardized mean difference.

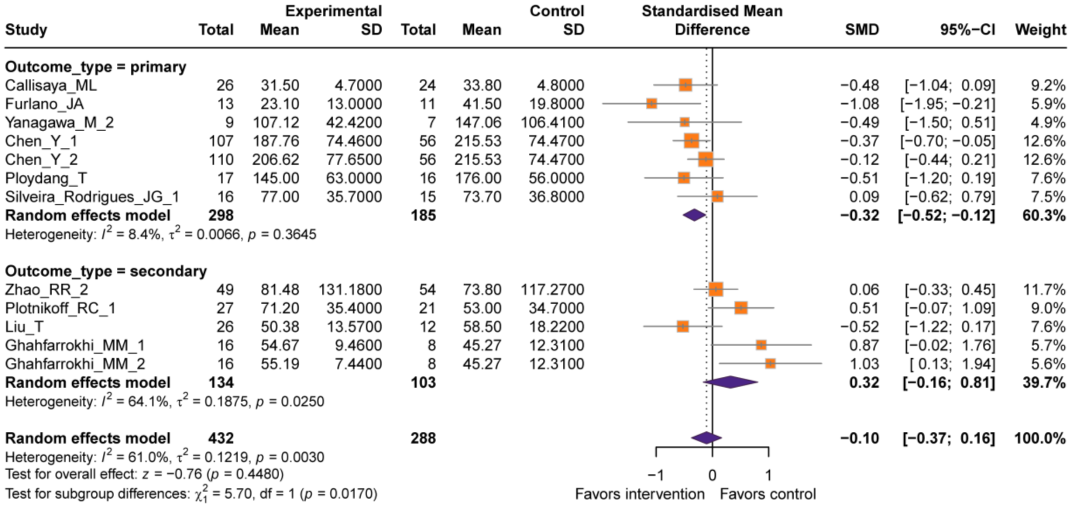

Figure S24. Sensitivity analysis of the effect of exercise on executive function, restricted to studies in which executive function was a prespecified primary outcome; 95%CI = 95% confidence interval; *df* = degree of freedom; I^2 = inconsistency between studies; SMD = standardized mean difference.

Study	Experimental Total	Experimental Mean	Experimental SD	Control Total	Control Mean	Control SD	SMD	95%−CI	Weight
Callisaya_ML	26	31.50	4.7000	24	33.80	4.8000	−0.48	[−1.04; 0.09]	12.0%
Furlano_JA	13	23.10	13.0000	11	41.50	19.8000	−1.08	[−1.95; −0.21]	5.3%
Yanagawa_M_2	9	107.12	42.4200	7	147.06	106.4100	−0.49	[−1.50; 0.51]	3.9%
Chen_Y_1	107	187.76	74.4600	56	215.53	74.4700	−0.37	[−0.70; −0.05]	31.2%
Chen_Y_2	110	206.62	77.6500	56	215.53	74.4700	−0.12	[−0.44; 0.21]	31.8%
Ploydang_T	17	145.00	63.0000	16	176.00	56.0000	−0.51	[−1.20; 0.19]	8.1%
Silveira_Rodrigues_JG_1	16	77.00	35.7000	15	73.70	36.8000	0.09	[−0.62; 0.79]	7.9%
**Random effects model**	**298**			**185**			**−0.32**	**[−0.52; −0.12]**	**100.0%**

Heterogeneity: $I^2 = 8.4\%$, $\tau^2 = 0.0066$, $p = 0.3645$
Test for overall effect: $z = -3.09$ ($p = 0.0020$)

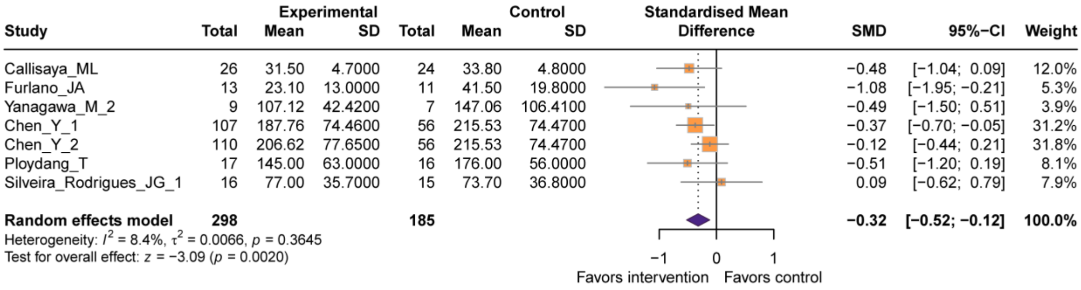

Table S1. The details of cognitive assessment tools of included studies

Study	Information of participants			Experimental Group Frequency, intensity	Duration	Outcomes
	Sample size and sex (male/female)	Mean age (years)	Form of Intervention			
Silveira-Rodrigues JG (2021)	31 participants with T2DM (No report)	63.6	Multicomponent exercise	3 days a week (22-28 min per session)<br>Intensity: No report	8 weeks	Digit Symbol Substitution – hits; Trail Making Test - part. A (s); 2.5 min (short-term memory);
Zhao RR (2022)	103 participants with T2DM (M: 51/F: 52)	67.9	Resistance exercise	3 days a week (60 min per session)<br>Intensity: 80% of 1 RM	48 weeks	Trail Making Test - part. B (s); Word list memory
Yanagawa M (2011)	16 female participants with T2DM (M: 11/ F: 5)	70.9	Aerobic exercise	4 days a week (45 min per session)<br>Intensity: No report	12 weeks	Trail Making Test - part. B (s); MMSE
Chen Y (2023)	328 participants with T2DM (M: 161/ F: 167)	67.5	Aerobic exercise	3 days a week (40 min per session)<br>Intensity: 50-70% of HR_{max} (heart rate max)	36 weeks	Digit Symbol Substitution Test score; MQ(Wechsler Memory Quotient); MOCA
Ploydang T (2023)	33 participants with T2DM (M: 12/ F: 21)	69.1	Aerobic exercise	3 days a week (40 min per session)<br>Intensity: 40-60% of HRR	12 weeks	Trail Making Test - part. B (s)
Furlano JA (2023)	24 participants with T2DM (M: 12/ F: 12)	68.7	Resistance exercise	3 days a week (60 min per session)<br>Intensity: 80% of 1 RM	24 weeks	The Victoria Stroop test (interference score C-D); Digit span

Liu T (2024)	38 participants with T2DM (M: 16/ F: 22)	56.8	Aerobic exercise	3 days a week (50 min per session)<br>Intensity: 40-59% of HRR	6 weeks	Processing speed; Working memory
Plotnikoff RC (2010)	38 participants with T2DM (M: 16/ F: 32)	54.5	Resistance exercise	3 days a week (40 min per session)<br>Intensity: No report	16 weeks	Task self-efficacy
Ghahfarrokhi MM (2024)	48 participants with T2DM (M: 17/ F: 31)	67.5	Multicomponent exercise	3 days a week (40 min per session)<br>Intensity: No report	6 weeks	Stroop (Number correct); MMSE
Shellington EM (2018)	25 participants with T2DM (M: 17/ F: 8)	68.6	Aerobic exercise	2 days a week (60 min per session)<br>Intensity: No report	24 weeks	Concentration-Rotations; Digit span
Ghodrati N (2022)	21 participants with T2DM (M: 0/ F: 21)	No report	Multicomponent exercise	3 days a week (50 min per session)<br>Intensity: No report	12 weeks	Digit Symbol Substitution Test score; MOCA
Callisaya ML (2017)	50 participants with T2DM (M: 26/ F: 24)	66.2	Multicomponent exercise	3 days a week (120 min per session)<br>Intensity: RPE 12-13	48 weeks	Trails B-A; RCF copy
Suh J (2019)	30 participants with T1DM (No report)	19.5	Multicomponent exercise	1 days a week (60min per session)<br>Intensity: No report	12 weeks	Digit span
Wang Y (2023)	82 participants with T2DM (M: 41/ F: 41)	66.9	Aerobic exercise	3 days a week (60min per session)<br>Intensity: No report	48 weeks	MMSE
Molina-Sotomayor E (2020)	107 participants with T2DM (M: 0/ F:107)	71.6	Aerobic exercise	3 days a week (60min per session)<br>Intensity: 40%-65%	24 weeks	MMSE

				VO_{2max}		
Sun W (2025)	128 participants with T2DM (M: 63/ F: 65)	70.6	Aerobic exercise	7 days a week (65min per session)<br>Intensity: No report	12 weeks	MMSE

RM: repetition maximum; HRR: heart rate reserve; RPE: rate of perceived exertion scale; VO_{2max}: maximum oxygen consumption; MMSE: Mini-Mental State Examination; MOCA: Montreal Cognitive Assessment

Table S2. The measurement tools for different outcomes

**Cognitive domains**	**Measurement tools of interests**
Global cognition	Mini-Mental State Examination (MMSE); The Montreal cognitive assessment (MOCA)
Executive function	Processing speed; Trial mark test (TMT-B, TMT-A); Task self-efficacy; The Victoria Stroop test (interference score C-D); Stroop (Number correct)
Memory	Working memory; 2.5 min (short-term memory); RCF copy; Digit span; MQ (Wechsler Memory Quotient); Word list memory
Attention	Digit Symbol Substitution – hits; Digit Symbol Substitution Test score; Concentration-Rotations
Motor performance	SF-12 physical composite score; 6-Minute Walk Test

Table S3. Methodological quality assessment of included studies

Items													
Study	1	2	3	4	5	6	7	8	9	10	11	Score[a]	Quality rating
Silveira-Rodrigues JG (2021)	1	1	0	1	0	0	0	1	0	1	1	6	High
Zhao RR (2022)	1	1	1	1	0	0	1	1	1	1	1	8	High
Yanagawa M (2011)	1	1	1	0	0	0	0	1	0	1	1	5	Moderate
Chen Y (2023)	1	1	1	1	0	0	1	1	1	1	1	8	High
Ploydang T (2023)	1	1	0	1	0	0	0	1	1	1	1	6	High
Furlano JA (2023)	1	1	0	1	0	0	0	1	1	1	1	6	High
Liu T (2024)	1	1	0	1	0	0	1	0	1	1	1	6	High
Plotnikoff RC (2010)	1	1	0	1	0	0	0	1	1	1	1	6	High
Ghahfarrokhi MM (2024)	1	1	1	1	0	0	1	1	1	1	1	8	High
Shellington EM (2018)	1	1	0	1	0	0	0	0	0	1	1	4	Moderate
Ghodrati N (2022)	1	1	0	1	0	0	0	0	1	1	1	5	Moderate
Callisaya ML (2017)	1	1	1	1	0	0	1	1	0	1	1	7	High

Suh J (2019)	1	0	0	0	0	0	0	1	0	1	1	3	Low
Wang Y (2023)	1	1	1	1	0	0	1	0	0	1	1	6	High
Molina-Sotomayor E (2020)	1	1	1	1	0	0	1	1	0	1	1	7	High
Sun W (2025)	1	1	0	1	0	0	0	1	0	1	1	5	Moderate

Items: (1) eligibility criteria; (2) randomization; (3) concealed allocation; (4) similarity at baseline; (5) subjects blinding; (6) blinding therapists; (7) assessors blinding; (8) one key outcome measured in > 85% of subjects; (9) intention to treat; (10) between-group statistical results for one key outcome; (11) measures of variability and point measures for one key outcome. [a] The total score on a 10-point scale is calculated according to the number of criteria met, with the exception that scale item 1 is not included in the computation of the overall score.

Table S4. Search strategy

PubMed (2025-12-27)	("Exercise"[Mesh] OR "Resistance Training"[Mesh] OR "Physical Conditioning, Human"[Mesh] OR "High-Intensity Interval Training"[Mesh] OR "Tai Ji"[Mesh] OR training[tiab] OR exercise[tiab] OR "resistance training"[tiab] OR plyometric*[tiab] OR "Tai Chi"[tiab] OR "weight exercise"[tiab] OR "weight training"[tiab] OR "weight lifting"[tiab] OR "resistance exercise"[tiab] OR "resistance therap*"[tiab] OR "strength training"[tiab] OR "strength exercise"[tiab] OR "strength therap*"[tiab] OR "strength workout"[tiab] OR "muscular training"[tiab] OR "muscle training"[tiab] OR "high intensity interval training"[tiab] OR HIIT[tiab]) AND ("Cognition"[Mesh] OR "Comprehension"[Mesh] OR "Memory"[Mesh] OR "Metacognition"[Mesh] OR "Perception"[Mesh] OR "Executive Function"[Mesh] OR "Attention"[Mesh] OR cognition*[tiab] OR cognitive*[tiab] OR neurocognit*[tiab] OR comprehension[tiab] OR memory[tiab] OR metacognit*[tiab] OR perception[tiab] OR "executive function*"[tiab] OR attention*[tiab]) AND ("Diabetes Mellitus"[Mesh] OR "Diabetes Mellitus, Type 1"[Mesh] OR "Diabetes Mellitus, Type 2"[Mesh] OR diabetes[tiab] OR diabetic[tiab] OR T2DM[tiab] OR T1DM[tiab] OR "type 2 diabetes"[tiab] OR "type 1 diabetes"[tiab])
Web of Science (2025-12-27)	TS=(training OR exercise OR "Tai Chi" OR "resistance training" OR "weight training" OR "weight lifting" OR "resistance exercise" OR "resistance therap*" OR "strength* training" OR "strength* exercise" OR "strength* therap*" OR "strength* workout" OR "power training" OR "power exercise" OR "musc* training" OR "high intensity interval training" OR HIIT) AND TS=(cognition* OR cognitive* OR neurocognit* OR comprehension OR memory OR metacognit* OR perception OR "executive function*" OR attention*) AND TS=(diabetes OR diabetic OR T2DM OR T1DM OR "type 2 diabetes" OR "type 1 diabetes")
Scopus (2025-12-27)	(TITLE-ABS-KEY(training OR exercise OR "Tai Chi" OR "weight exercise" OR "weight training" OR "weight lifting" OR "resistance exercise" OR "resistance therap*" OR "strength* training" OR "strength* exercise" OR "strength* therap*" OR "strength* workout" OR "power training" OR "power exercise" OR "musc* training" OR "high intensity interval training" OR HIT OR HIIT)) AND (TITLE-ABS-KEY(cognition* OR cognitive* OR neurocognit* OR comprehension OR memory OR

	metacognit* OR perception OR "executive function*" OR attention*)) AND (TITLE-ABS-KEY(diabetes OR diabetic OR T2DM OR T1DM OR "type 2 diabetes" OR "type 1 diabetes"))
EMBASE (2025-12-27)	(exp exercise/ OR exp training/ OR exp resistance training/ OR exp Tai Chi/ OR exp "high intensity interval training"/ OR training.ti,ab,kw. OR exercise.ti,ab,kw. OR "Tai Chi".ti,ab,kw. OR "weight exercise".ti,ab,kw. OR "weight training".ti,ab,kw. OR "weight lifting".ti,ab,kw. OR "resistance exercise".ti,ab,kw. OR "resistance therap*".ti,ab,kw. OR "strength* training".ti,ab,kw. OR "strength* exercise".ti,ab,kw. OR "strength* therap*".ti,ab,kw. OR "strength* workout".ti,ab,kw. OR "power training".ti,ab,kw. OR "power exercise".ti,ab,kw. OR "musc* training".ti,ab,kw. OR "high intensity interval training".ti,ab,kw. OR HIIT.ti,ab,kw. OR HIT.ti,ab,kw.) AND (exp cognition/ OR exp comprehension/ OR exp memory/ OR exp metacognition/ OR exp perception/ OR exp "executive function"/ OR exp attention/ OR cognition*.ti,ab,kw. OR cognitive*.ti,ab,kw. OR neurocognit*.ti,ab,kw. OR comprehension.ti,ab,kw. OR memory.ti,ab,kw. OR metacognit*.ti,ab,kw. OR perception.ti,ab,kw. OR "executive function*".ti,ab,kw. OR attention*.ti,ab,kw.) AND (exp diabetes mellitus/ OR exp diabetic neuropathy/ OR exp diabetic angiopathy/ OR diabetes.ti,ab,kw. OR diabetic.ti,ab,kw. OR T2DM.ti,ab,kw. OR T1DM.ti,ab,kw. OR "type 2 diabetes".ti,ab,kw. OR "type 1 diabetes".ti,ab,kw.)
SPORTDiscus (2025-12-27)	(DE "RESISTANCE training" OR DE "ISOMETRIC exercise" OR DE "WEIGHT training" OR DE "PLYOMETRICS" OR TX training OR TX exercise OR TX "Tai Chi" OR TX "weight exercise" OR TX "weight training" OR TX "weight lifting" OR TX "resistance exercise" OR TX "resistance therap*" OR TX "strength* training" OR TX "strength* exercise" OR TX "strength* therap*" OR TX "strength* workout" OR TX "power training" OR TX "power exercise" OR TX "musc* training" OR TX "high intensity interval training" OR TX HIIT) AND (DE "COGNITION" OR DE "COGNITIVE ability" OR DE "MEMORY" OR DE "PERCEPTION" OR DE "ATTENTION" OR TX cognition* OR TX cognitive* OR TX neurocognit* OR TX comprehension OR TX memory OR TX metacognit* OR TX perception OR TX "executive function*" OR TX attention*) AND (DE "DIABETES" OR DE "DIABETES mellitus" OR TX diabetes OR TX diabetic OR TX T2DM OR TX T1DM OR TX "type 2 diabetes" OR TX "type 1 diabetes")

PRISMA 2020 Checklist

Table S5. Check list

Section and Topic	Item #		Location where item is reported
**TITLE**			
Title	1	Identify the report as a systematic review.	Optimizing Exercise Prescriptions for Cognitive Subdomains in Diabetes: A Systematic Review and Meta-Analysis of Dose-Response Variables
**ABSTRACT**			
Abstract	2	See the PRISMA 2020 for Abstracts checklist.	Abstract
**INTRODUCTION**			
Rationale	3	Describe the rationale for the review in the context of existing knowledge.	Introduction（paragraph 1-3）
Objectives	4	Provide an explicit statement of the objective(s) or question(s) the review addresses.	Introduction（paragraph 4）
**METHODS**			
Eligibility criteria	5	Specify the inclusion and exclusion criteria for the review and how studies were grouped for the syntheses.	2.2. Inclusion and Exclusion Criteria
Information sources	6	Specify all databases, registers, websites, organisations, reference lists and other sources searched or consulted to identify studies. Specify the date when each source was last searched or consulted.	2.1. Overview
Search strategy	7	Present the full search strategies for all databases, registers and websites, including any filters and limits used.	2.1. Overview
Selection process	8	Specify the methods used to decide whether a study met the inclusion criteria of the review, including how many reviewers screened each record and each report retrieved, whether they worked independently, and if applicable, details of automation tools used in the process.	2.3. Data Extraction and coding
Data collection process	9	Specify the methods used to collect data from reports, including how many reviewers collected data from each report, whether they worked independently, any processes for obtaining or confirming data from study investigators, and if applicable, details of automation tools used in the process.	2.3. Data Extraction and coding
Data items	10a	List and define all outcomes for which data were sought. Specify whether all results that were compatible with each outcome domain in each study were sought (e.g. for all measures, time points, analyses), and if not, the methods used to decide which results to collect.	2.2. Inclusion and Exclusion Criteria
	10b	List and define all other variables for which data were sought (e.g. participant and intervention characteristics, funding sources). Describe any assumptions made about any missing or unclear information.	2.3. Data Extraction and coding
Study risk of bias assessment	11	Specify the methods used to assess risk of bias in the included studies, including details of the tool(s) used, how many reviewers assessed each study and whether they worked independently, and if applicable, details of automation tools used in the process.	2.4. Quality and Risk of Bias Assessments
Effect measures	12	Specify for each outcome the effect measure(s) (e.g. risk ratio, mean difference) used in the synthesis or presentation of results.	2.5. Statistical Analysis
Synthesis methods	13a	Describe the processes used to decide which studies were eligible for each synthesis (e.g. tabulating the study intervention characteristics and comparing against the planned groups for each synthesis (item #5)).	3.2. Study Characteristics
	13b	Describe any methods required to prepare the data for presentation or synthesis, such as handling of missing summary statistics, or data conversions.	2.5. Statistical Analysis

PRISMA 2020 Checklist

Section and Topic	Item #		Location where item is reported
	13c	Describe any methods used to tabulate or visually display results of individual studies and syntheses.	2.5. Statistical Analysis
	13d	Describe any methods used to synthesize results and provide a rationale for the choice(s). If meta-analysis was performed, describe the model(s), method(s) to identify the presence and extent of statistical heterogeneity, and software package(s) used.	2.5. Statistical Analysis
	13e	Describe any methods used to explore possible causes of heterogeneity among study results (e.g. subgroup analysis, meta-regression).	3.3. Methodological quality and Risk of bias
	13f	Describe any sensitivity analyses conducted to assess robustness of the synthesized results.	3.3. Methodological quality and Risk of bias
Reporting bias assessment	14	Describe any methods used to assess risk of bias due to missing results in a synthesis (arising from reporting biases).	2.4. Quality and Risk of Bias Assessments; 3.3. Methodological quality and Risk of bias
Certainty assessment	15	Describe any methods used to assess certainty (or confidence) in the body of evidence for an outcome.	2.4. Quality and Risk of Bias Assessments; 2.5. Statistical Analysis
**RESULTS**			
Study selection	16a	Describe the results of the search and selection process, from the number of records identified in the search to the number of studies included in the review, ideally using a flow diagram.	3.1. Literature Search
	16b	Cite studies that might appear to meet the inclusion criteria, but which were excluded, and explain why they were excluded.	3.1. Literature Search
Study characteristics	17	Cite each included study and present its characteristics.	3.2. Study Characteristics
Risk of bias in studies	18	Present assessments of risk of bias for each included study.	2.4. Quality and Risk of Bias Assessments
Results of individual studies	19	For all outcomes, present, for each study: (a) summary statistics for each group (where appropriate) and (b) an effect estimate and its precision (e.g. confidence/credible interval), ideally using structured tables or plots.	3.4 Effect measures (table3-6)
Results of syntheses	20a	For each synthesis, briefly summarise the characteristics and risk of bias among contributing studies.	2.4. Quality and Risk of Bias Assessments
	20b	Present results of all statistical syntheses conducted. If meta-analysis was done, present for each the summary estimate and its precision (e.g. confidence/credible interval) and measures of statistical heterogeneity. If comparing groups, describe the direction of the effect.	3.4 Effect measures
	20c	Present results of all investigations of possible causes of heterogeneity among study results.	3.4 Effect measures
	20d	Present results of all sensitivity analyses conducted to assess the robustness of the synthesized results.	3.4 Effect measures
Reporting biases	21	Present assessments of risk of bias due to missing results (arising from reporting biases) for each synthesis assessed.	3.3. Methodological quality and Risk of bias
Certainty of evidence	22	Present assessments of certainty (or confidence) in the body of evidence for each outcome assessed.	3.4 Effect measures
**DISCUSSION**			
Discussion	23a	Provide a general interpretation of the results in the context of other evidence.	Discussion (paragraph 1)

PRISMA 2020 Checklist

Section and Topic	Item #		Location where item is reported
	23b	Discuss any limitations of the evidence included in the review.	4.5. Limitations
	23c	Discuss any limitations of the review processes used.	4.5. Limitations
	23d	Discuss implications of the results for practice, policy, and future research.	4. Discussion
**OTHER INFORMATION**			
Registration and protocol	24a	Provide registration information for the review, including register name and registration number, or state that the review was not registered.	PROSPERO(CRD420251046731)
	24b	Indicate where the review protocol can be accessed, or state that a protocol was not prepared.	PROSPERO
	24c	Describe and explain any amendments to information provided at registration or in the protocol.	N/A
Support	25	Describe sources of financial or non-financial support for the review, and the role of the funders or sponsors in the review.	N/A
Competing interests	26	Declare any competing interests of review authors.	The author of the commentary has no conflicts of interest
Availability of data, code and other materials	27	Report which of the following are publicly available and where they can be found: template data collection forms; data extracted from included studies; data used for all analyses; analytic code; any other materials used in the review.	N/A

From: Page MJ, McKenzie JE, Bossuyt PM, Boutron I, Hoffmann TC, Mulrow CD, et al. The PRISMA 2020 statement: an updated guideline for reporting systematic reviews. BMJ 2021;372:n71. doi: 10.1136/bmj.n71

For more information, visit: http://www.prisma-statement.org/
